# Wash‐free fluorescent tools based on organic molecules: Design principles and biomedical applications

**DOI:** 10.1002/EXP.20230094

**Published:** 2024-06-28

**Authors:** Jingyun Tan, Chunfei Wang, Zhangjun Hu, Xuanjun Zhang

**Affiliations:** ^1^ Faculty of Health Sciences University of Macau Macau China; ^2^ Department of Pharmacology School of Pharmacy Wannan Medical College Wuhu China; ^3^ Department of Physics, Chemistry and Biology (IFM) Linköping University Linköping Sweden; ^4^ MOE Frontiers Science Centre for Precision Oncology University of Macau Macau China

**Keywords:** biomedical applications, design principles, molecular fluorescent tools, wash‐free

## Abstract

Fluorescence‐assisted tools based on organic molecules have been extensively applied to interrogate complex biological processes in a non‐invasive manner with good sensitivity, high resolution, and rich contrast. However, the signal‐to‐noise ratio is an essential factor to be reckoned with during collecting images for high fidelity. In view of this, the wash‐free strategy is proven as a promising and important approach to improve the signal‐to‐noise ratio, thus a thorough introduction is presented in the current review about wash‐free fluorescent tools based on organic molecules. Firstly, generalization and summarization of the principles for designing wash‐free molecular fluorescent tools (WFTs) are made. Subsequently, to make the thought of molecule design more legible, a wash‐free strategy is highlighted in recent studies from four diverse but tightly binding aspects: (1) special chemical structures, (2) molecular interactions, (3) bio‐orthogonal reactions, (4) abiotic reactions. Meanwhile, biomedical applications including bioimaging, biodetection, and therapy, are ready to be accompanied by. Finally, the prospects for WFTs are elaborated and discussed. This review is a timely conclusion about wash‐free strategy in the fluorescence‐guided biomedical applications, which may bring WFTs to the forefront and accelerate their extensive applications in biology and medicine.

## Graphical abstract

In this review, the design principles for wash‐free molecular fluorescent tools are highlighted in four diverse details. Meanwhile, biomedical applications including wash‐free fluorescent imaging of organelles, cellular microenvironment, biomacromolecules, and small species, as well as therapy and potential drug delivery, are also emphasized. This review may bring wash‐free molecular fluorescent tools to the forefront, as well as expand the applications in biology and medicine.

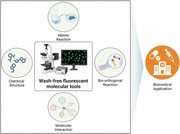

## 1 INTRODUCTION

**SCHEME 1:**
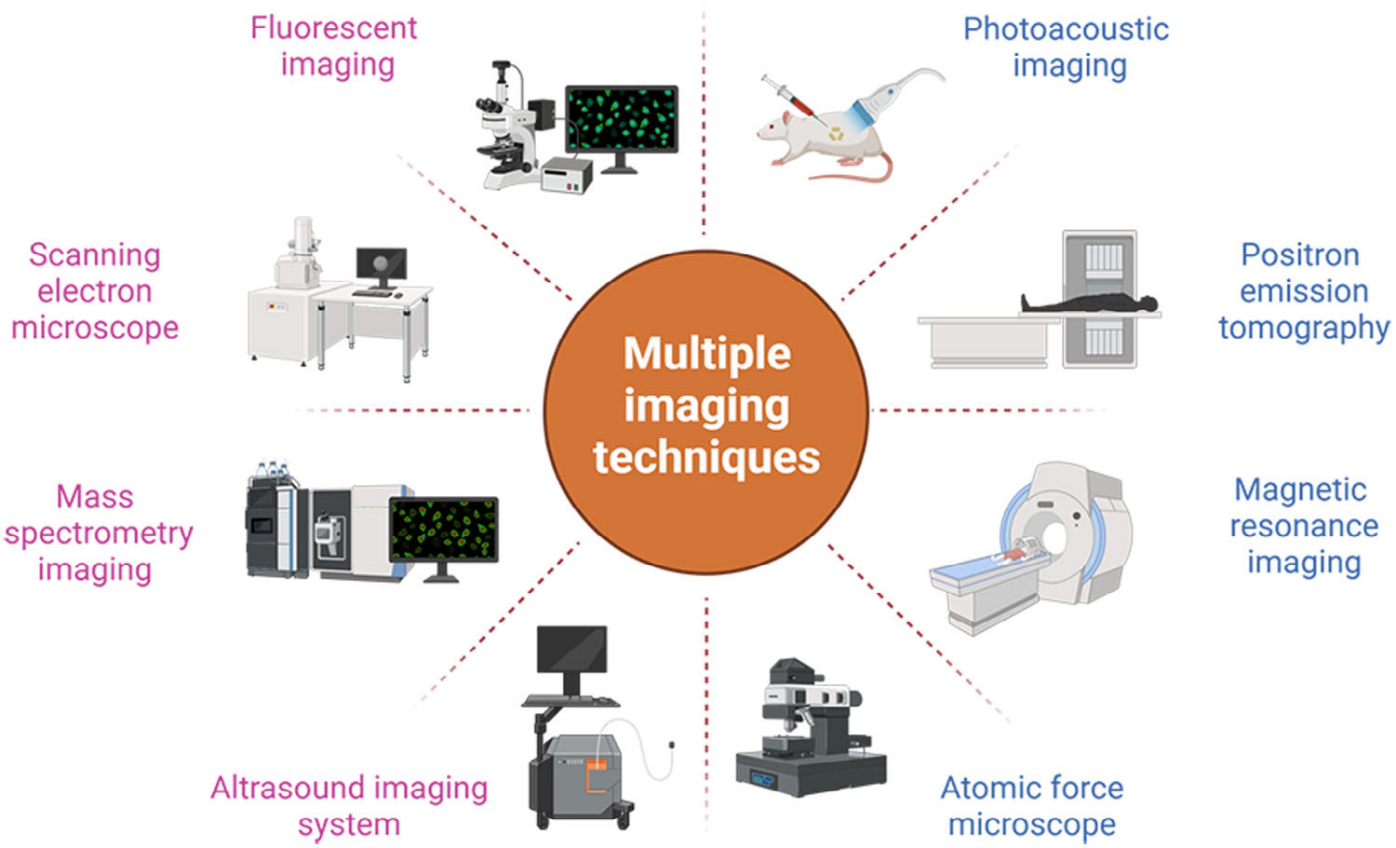
Different kinds of imaging techniques developed so far.

Biological imaging is of immense importance either in the diagnosis and therapy of disease or in fundamental biology research. It provides infinite possibilities to visualize the inside of the whole body in depth involving organs, tissues, and cells, which totally depends on different initiated conditions such as light, sound wave, X‐ray, magnetism, electron beam, positron beam, and ion beam. As illustrated in Scheme 1, different technologies are emerging subsequently as required, such as fluorescent imaging,^[^ ^1^ ^]^ ultrasound and photoacoustic imaging,^[^ ^2^ ^]^ computed tomography,^[^ ^3^ ^]^ mass spectrometry imaging,^[^ ^4^ ^]^ magnetic resonance imaging,^[^ ^5^ ^]^ atomic force microscope,^[^ ^6^ ^]^ positron emission tomography,^[^ ^7^ ^]^ and so forth. Among these techniques, fluorescent imaging has drawn enormous attention, because of the merit of obtaining details in a non‐invasive manner with good sensitivity, high resolution, easily operation and excellent contrast. Currently, fluorescent imaging benefits largely from the support of rapidly developing technology of microscope, and further be strongly fueled by improvements in fluorescent tools and image analysis methods.^[^ ^8^ ^]^ As a result, fluorescence‐guided biological studies are economical, easily accessible, and widely adopted.

The fluorescent signal distribution is measured under exciting illumination and finally collecting as a digital image of the sample. As a result, fluorescent imaging is especially suitable for the research of physiological processes at a molecular level, which allowing the visualization of cellular and sub‐cellular activities within tiny spatial resolutions (from nanometers to micrometers). In recent years, fluorescent nanomaterials have been extensively applied in therapy,^[^ ^9^ ^]^ biodetection, and biosensors.^[^ ^10^ ^]^ Fluorescent nanomaterials have achieved success in many high‐level studies, but their scale, uncertain concentration, and poor reproducibility limited their development. Notably, organic fluorescent tools, like dyes, probes, and intelligent photosensitizers, endow samples be labeled via distinct colors for the application of fluorescent imaging‐related studies. The superiorities, such as definite chemical structure, tunable concentration, and excellent reproducibility, make organic fluorescent tools well‐applied, even be developed as commercial tools. To date, their studied topic covers the function and activity of various organelles (like the cell membrane, cell nucleus, mitochondria, lysosomes, and endoplasmic reticulum), homeostatic cellular microenvironment (such as polarity, viscosity, pH, and temperature), subcellular localization and dynamics of biomacromolecules (proteins, hormones, and nucleic acids), along with small functional species (amino acids, ATP, oxidative/antioxidative species, metal ions, and anions). Besides, fluorescence‐guided surgery and photodynamic therapy for cancer have also emerged as a burgeoning area in recent years.

Although fluorescent tools have flexibly been applied in biology and medicine, signal‐to‐noise ratio is a crucial factor for high‐quality imaging. In respect of fluorescent imaging, the noise comes from auto‐fluorescence of the background and wandering fluorophores (unfixed or unlabeled fluorescent dyes and probes). Lots of emphases have been put on the way to decrease the interference from auto‐fluorescence, rather than those from the emitting tools themselves are often overlooked. In general, the most common approach to reduce the interference is removing emitting residual as much as possible by washing procedures before final image acquisition. Nevertheless, the repeating washing procedures is often time‐consuming which may hinder real‐time in vivo imaging and continuous monitoring of targets. Moreover, the washing process may change the environment of the specimen, which might introduce deviations towards the acquired results. Consequently, it inevitably reduces the credibility of imaging data. Aiming especially at those drawbacks, fluorescent tools based on wash‐free is a more promising technique by virtue of being exempt from washing procedures during the final acquisition process, which can not only improve the signal‐to‐noise ratio but also facilitate the real‐time trace of the target biological process in vivo, thus arousing increasing interests from researchers, especially in biology and medicine. Here, nano‐scale carbon dots have been developed as hydrogen‐bond‐induced emission for wash‐free nucleus imaging, as well as nitrogen‐doped carbon dots.^[^ ^11^ ^]^ Besides, copper nanosheet has also been reported for wash‐free fluorescence imaging of cancer cells.^[^ ^12^ ^]^ Due to the shortcomings of nanomaterials mentioned above, nano‐scale fluorescent tools based on wash‐free are not well exploited and applied.

In fact, wash‐free comes from the concept of fluorogenicity realized by organic dyes/probes, which can be dated back to the study by Kikuchi and co‐workers.^[^ ^13^ ^]^ The demand for washing free is originally derived from protein labeling technologies which are utilized for visualizing proteins of interest (POIs). Therefore, most of the pioneer works are regarding to the construction and application of POI‐tag systems owning fluorogenic features. Kikuchi and coworkers have made heroic efforts to protein labeling and the related achievements have been successfully overviewed.^[^ ^13^, ^14^ ^]^ They highlighted the wash‐free strategy, and briefly summarized the chemical principles for the design of various POI‐tag systems and the advance in functional protein labeling/imaging. So far, lots of fluorogenic tools have been prepared to be applicable for wash‐free imaging. Additionally, Schultz and coworkers emphasized the power of fluorogenic probes and gave a hint about the capacity of no‐wash labeling application in a short highlight.^[^ ^15^ ^]^ Meanwhile, Raines and coworkers discussed the wash‐free strategy in different fluorescent probes activated by enzymes for in vivo imaging and summarized the principle of fluorescence masking methods, such as fluorescence quenching, group masking, and other chemical modifications.^[^ ^16^ ^]^ In 2017, Klymchenko reviewed the design principles of fluorogenic probes directed at environment‐sensitive issues which were summarized with good classification, while the bright future of the wash‐free concept is also revealed.^[^ ^17^ ^]^ However, most fluorogenicity topics did not highlight the tremendous potential for wash‐free enough and some newly emerging wash‐free strategies are also not covered in fluorescent imaging.

**SCHEME 2:**
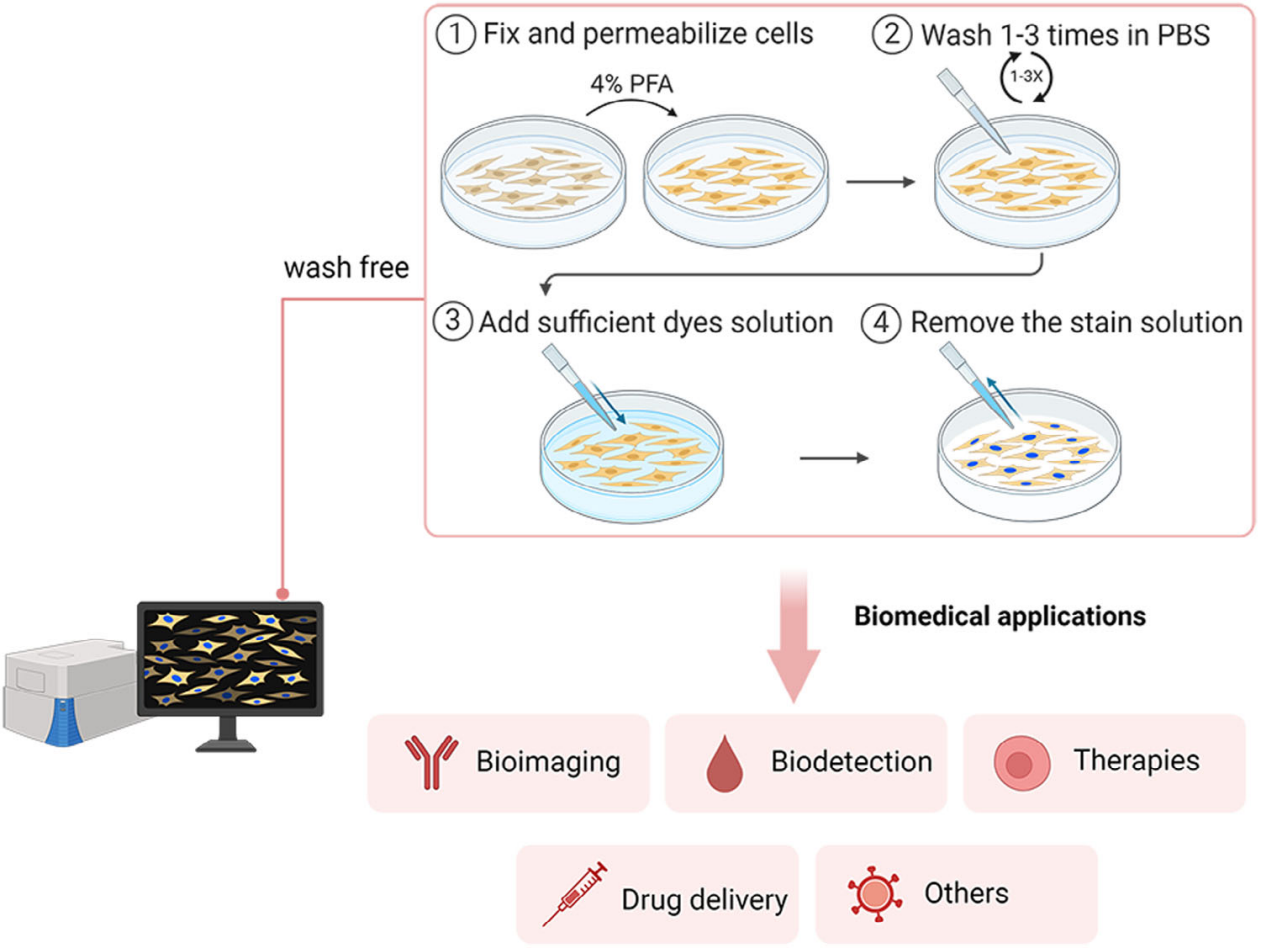
Schematic illustration of wash‐free strategy for biomedical applications.

As the effective strategy for improving signal‐to‐noise ratio and realizing noninvasive fluorescent imaging, we endeavor to systematically conclude and introduce fluorescent tools with wash‐free capacity in this review (Scheme 2). Firstly, we summarize the design principles of wash‐free molecular fluorescent tools (WFTs) based on the special chemical structure of organic molecules, molecular interactions, bio‐orthogonal reactions, and abiotic reactions. Secondly, attention to biomedical applications is not merely focused on protein‐related research, but on a broad scope which includes wash‐free fluorescent imaging of organelles, cellular microenvironment, biomacromolecules, and small species, as well as therapy and potential drug delivery. Thirdly, future development and trends are looked forward to. The overview may lead timely promotion of biomedical applications based on wash‐free and hopefully facilitate the development of fluorescent tools based on organic molecules for wash‐free in future biomedical studies.

## 2 DESIGN PRINCIPLES OF MOLECULAR TOOLS FOR WASH‐FREE

The essential aspect of wash‐free fluorescent imaging is organic dyes or probes with significant turn‐on fluorescence changes after being functioned. The fluorescent signal is switchable in a tunable way, namely it can only light up by specific external stimulus or particular inside environments, even specific reactions, otherwise keeps quenching. Herein, we highlight already used design principles of WFTs with four aspects. Firstly, the design principles for WFTs are elaborated from a chemical perspective, which were rationally designed on the ground of AIE moieties, strong donor‐acceptor mode, and structure isomerization. This strategy is mostly committed to monitoring physiological polarity. Secondly, this classification criteria are that the formation of covalent bonds is not involved during the light‐up process. It contains four categories of molecular interaction: (1) hydrogen bonding interaction, (2) hydrophobic interaction, (3) π–π interaction, (4) van der Waals forces interaction. Although noncovalent bonds are easily attacked, this interaction play indispensable roles in wash‐free fluorescent imaging. Thirdly, a bio‐orthogonal reaction can also accomplish ideal wash‐free through intelligent modification, which mainly depends on the click reactions to turn on fluorescence. Fourthly, the abiotic reaction is a kind of extrinsic reaction by virtue of intrinsic species/environment in living systems to complete fluorescence light‐up as a wash‐free strategy. Ultimately, the recent advances in the design of WFTs are summarized and analyzed based on the above classification and legibly present the strategy of molecule design in the whole review.

## 3 WASH‐FREE MOLECULAR TOOLS BASED ON CHEMICAL STRUCTURE

### 3.1 Fluorophores based on AIE

Tang and co‐workers took the lead in proposing AIE in 2001 with the report of 1‐methyl‐1,2,3,4,5‐pentaphenylsilole.^[^ ^18^ ^]^ Since the concept of AIE was proposed, AIE luminogens (AIEgens) have been extensively developed, and also universally applied in living systems as molecular tools due to the special emission enhancement by aggregation. Here, AIEgens are characterized as non‐emissive when fully dispersed in solution, while turning on the emission when they are in the aggregated or solid state ascribing to the restriction of intramolecular motion.^[^ ^19^ ^]^ As well known, AIEgens can be easily aggregated in the biological environment which is of significant importance to turn‐on fluorescence as wash‐free. The use of AIEgens for biological imaging is not perfect, such as concentration dependence and non‐specific aggregation, but this strategy is still appropriate for fluorescence imaging with wash‐free. This section emphasizes the AIEgens for visualizing different subcellular structures or biological processes in a wash‐free manner.

#### 3.1.1 Cell membrane‐specific AIEgens

The cell membrane is the first barrier for exchanging and circulating of substances inside and outside cells. Cell membrane protects cells from harsh environment around, ensuring the cellular environmental homeostasis and enabling a number of biological processes to orderly operate.^[^ ^20^ ^]^ Generally, the cell membrane is basically made up of lipids, carbohydrates, and proteins in prokaryotic and eukaryotic cells, but a slight difference in the constituents of membrane. Here, bacteria and fungi are taken as examples. The most significant discrimination is that the peptidoglycan of the cell wall in Gram‐negative (G^−^) bacteria is less than that in Gram‐positive (G^+^) bacteria, while the structure of out membrane is only presented in G^−^ bacteria. Besides, cell membrane of bacteria consists of lipids and proteins about the same proportion, while the variation of phospholipids such as the length, charge, and saturation, lead to the significant distinction in different bacteria.^[^ ^21^ ^]^ Nevertheless, the cell membrane of fungi is abundant in diverse glycerophospholipids, sphingolipids, and sterols, while typically owing ergosterol as the main component comparing with that of mammalian cells containing cholesterol.^[^ ^22^ ^]^ In response to the different composition of cell membrane, it is possible to distinguish mammalian cells, bacteria, and fungi.

AIEgens can display strong fluorescence after the restriction of intramolecular motions in a specific environment. Amphipathic AIEgens have been successfully established for distinguishing cell membranes because they can bind with the lipophilic moiety of the phospholipid bilayer in cell membrane resulting in efficient insertion to restrict intramolecular motions and then turning on strong emission. In other words, the unconformity of phospholipid content between G^+^ and G^−^ bacteria in the cell membrane makes the AIEgens display different fluorescent properties, due to the different capabilities to restrict intramolecular motions. Presently, AIEgens are widely employed in the bioimaging of cell membranes in the mammalian cells and pathogens, which is the simplest wash‐free strategy.

**FIGURE 1:**
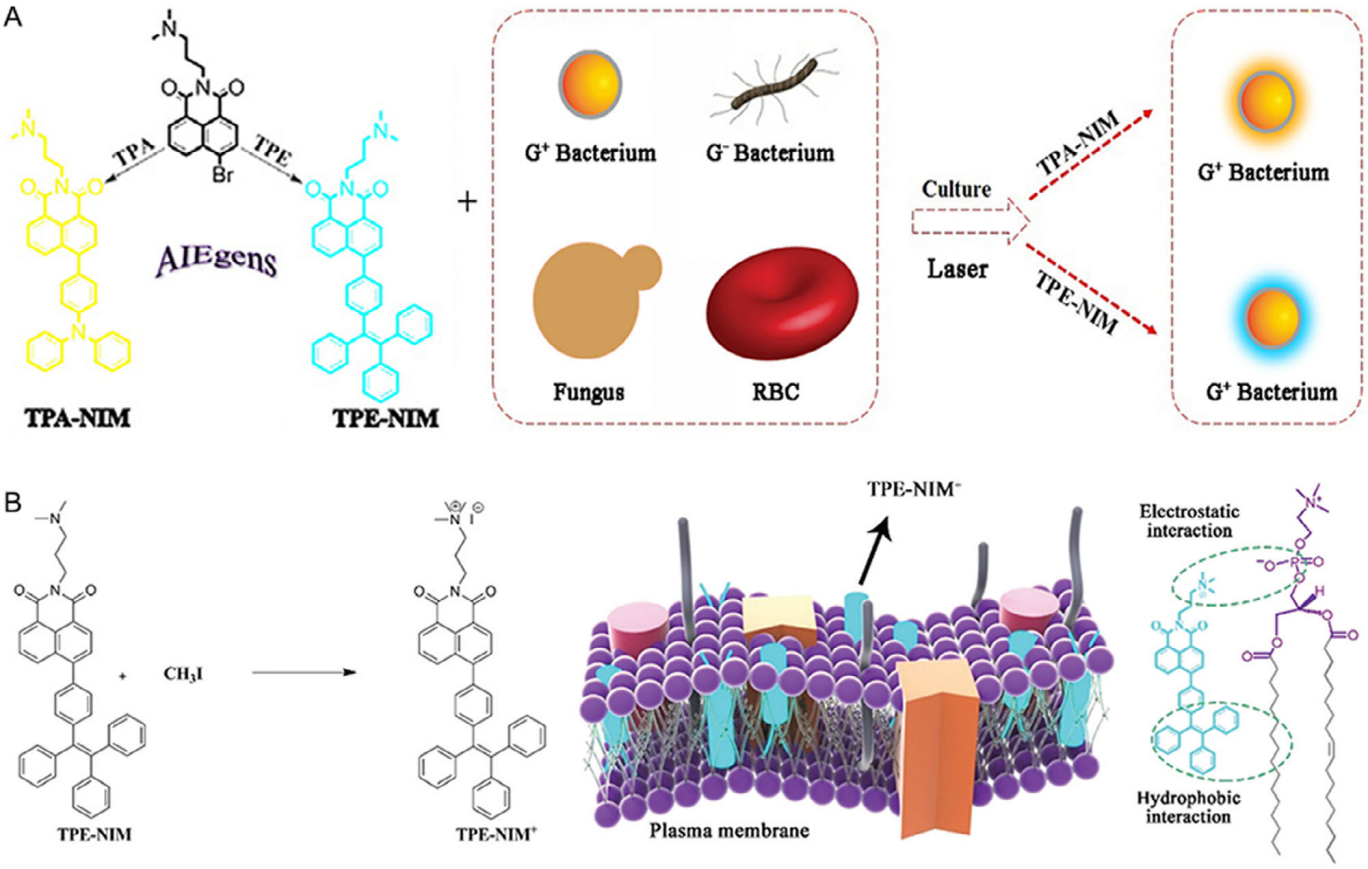
Naphthalimide moiety with AIE feature developed for wash‐free manner. (A) Molecular structures of AIEgens and a general representation of their staining for different pathogens (bacteria and fungus). Reproduced with permission.^[^ ^23^ ^]^ Copyright 2021, The Royal Society of Chemistry. (B) Molecular structures of TPE–NIM and TPE–NIM^+^ and the proposed mechanism of plasma membrane staining by TPE–NIM^+^. Reproduced with permission.^[^ ^24^ ^]^ Copyright 2021, The Royal Society of Chemistry.

Two bioprobes (TPE‐NIM and TPA‐NIM) were synthesized on the ground of naphthalimide moiety with AIE feature by Lu and co‐workers,^[^ ^23^ ^]^ which could selectively stain G^+^ bacteria through a wash‐free procedure but not G^−^ bacteria and Fungi (Figure 1A). These two AIE‐based bioprobes exhibited highly selective and wash‐free ability toward G^+^ bacteria, which was because different components in the cell membrane induced the distinction of electrostatic attraction and hydrophobic interaction. These two bioprobes can not only successfully distinguish the G^+^ bacteria from G^−^ bacteria and fungi, but also quickly track *Staphylococcus aureus* in the suspension of red blood cells without inducing significant hemolysis. The early detection of pathogens is necessary for the rational use of antibiotics; thus, this study is of guiding significance to detect pathogen infections in the clinic, especially G^+^ bacteria. As an extension of this study, Lu and co‐workers subsequently constructed a high photostable molecule regarding an AIEgen, tetraphenylethylene–naphthalimide^+^ (TPE–NIM^+^).^[^ ^24^ ^]^ Here, the positively charged quaternary amine endows TPE–NIM^+^ comforting features, such as ultrafast staining and long retention time at the cell membrane due to the stronger electrostatic attraction and hydrophobic interaction than that of TPE‐NIM and TPA‐NIM. Furthermore, the positively charged quaternary amine and hydrophobic moiety enable to stain the cell membrane with wash‐free properties in different cell lines (Figure 1B). More importantly, TPE–NIM^+^ displayed a more rapid targeting ability to cell membranes than that of commercial DiD and WGA‐Alexa Fluor 488, while exhibiting higher photostability than that of commercial dyes (WGA‐Alexa Fluor 488) after continuous excitation for 10 min. This superiority of TPE–NIM^+^ is significant in describing the morphology of living cells and future visualization of cell membrane engineering. Unfortunately, the short emissive wavelength is the main shortcomings of tetraphenylethylene and triphenylamine moiety, thus limiting the application of TPE‐NIM, TPA‐NIM, and TPE‐NIM^+^ in deep infection.

In addition, an amphiphilic C6‐BD was elaborately engineered and prepared by Tang and co‐workers with AIE properties and the characteristics of excited state intramolecular proton transfer (ESIPT),^[^ ^25^ ^]^ which was successfully employed to visualize cell membrane in a wash‐free manner. C6‐BD displayed turn‐on fluorescence to image G^+^ bacteria, G^−^ bacteria, and fungi, which benefited from the strong affinity to the membrane structure because of electrostatic attraction and hydrophobic interaction. Furthermore, C6‐BD also possessed antibacterial efficiency after light irradiation due to the generation of reactive oxygen species (ROS) during the photodynamic procedure. The significance of this study is that AIE‐based wash‐free molecular tools also display antibacterial and antifungal activity, which is potential for future chemotherapy. This study is also an excellent paradigm of wash‐free molecular tools in the application of theranostics.

Meanwhile, Chen and co‐workers developed a near‐infrared TBTCP with active AIE capacity, to monitor phagocytosis in a wash‐free process not only by fluorescent imaging but also by fluorescence lifetime imaging (FLTI).^[^ ^26^ ^]^ As well known, FLTI is not totally dependent on the local concentration of fluorophores but on its molecular environment,^[^ ^27^ ^]^ thus providing renewable quantitative measurements over time. In the current study, TBTCP was successfully applied to label cellular membranes by the fluorescence lifetime changes with rapid staining, high signal‐to‐noise ratio, and excellent photostability. In addition, this functional tool of TBTCP effectively monitors the dynamics of macrophage phagocytosis in complicated membrane physiology, such as cocultured with bacteria and in pro‐inflammatory responses and exposed to different sizes of silica particles. This may effectively assist the exploration of phagocytic function‐related studies. In short, the functional TNTCP for FLTI devised in the current study is a good example of wash‐free guided bioimaging by overcoming the disturbance of local concentration of fluorophores. Taking advantage of a restriction of intramolecular motions, Wang and co‐workers designed and synthesized Car‐py with AIE property,^[^ ^28^ ^]^ for fluorescence visualizing cell membranes in vitro and in vivo with a wash‐free procedure. Here, Car‐py presented excellent specificity and ultrafast staining towards cell membrane with strong electrostatic attraction and hydrophobic interaction, thus working well in many complicated living systems, including 3D cell models, mouse brains, and zebrafish. However, it is unavailable for in vivo imaging in deep tissue due to the short emissive wavelength. Moreover, Car‐py performed more stable photobleaching than that of commercial dyes, Dil, which is beneficial to the in vivo wash‐free imaging and may launch a challenge to commercially available dyes.

**FIGURE 2:**
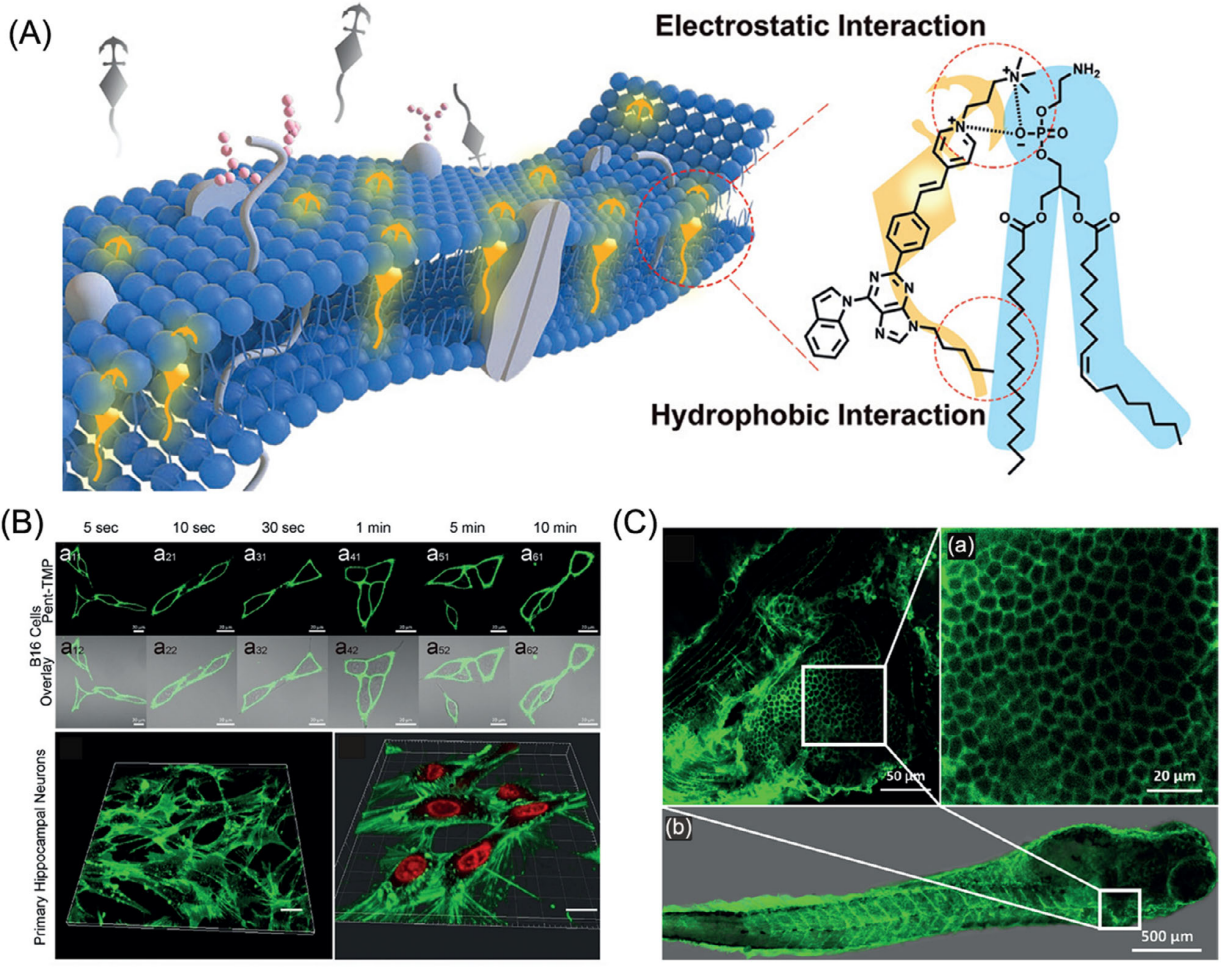
AIE‐based fluorescent molecule for wash‐free imaging of cell membrane. (A) Schematic of probes and plasma membrane (left). A single probe and phospholipid molecule to illustrate the proposed labeling strategy (right). (B) B16 cell stained for different times with Pent‐TMP. The 3D reconstructed image of primary hippocampal neurons stained with (a,b) solo Pent‐TMP and (c) Pent‐TMP with NucRed, (d) Z‐stack images of the 3D spheroid from 0–120 mm. (e) Cross‐sectional images of the *x*‐ and *y*‐axes. (f) The *z*‐axis cut layer at 60 mm. Scale bar = 20 µm. (C) Image of zebrafish gills. (a) The enlarged view of the surface of zebrafish gills with single‐cell resolution. (b) The pattern of zebrafish. Reproduced with permission.^[^ ^19c^ ^]^ Copyright 2020, Wiley. [Correction added on 3rd July 2024, after first online publication: Figure 2 was replaced with correct one.]

**FIGURE 3:**
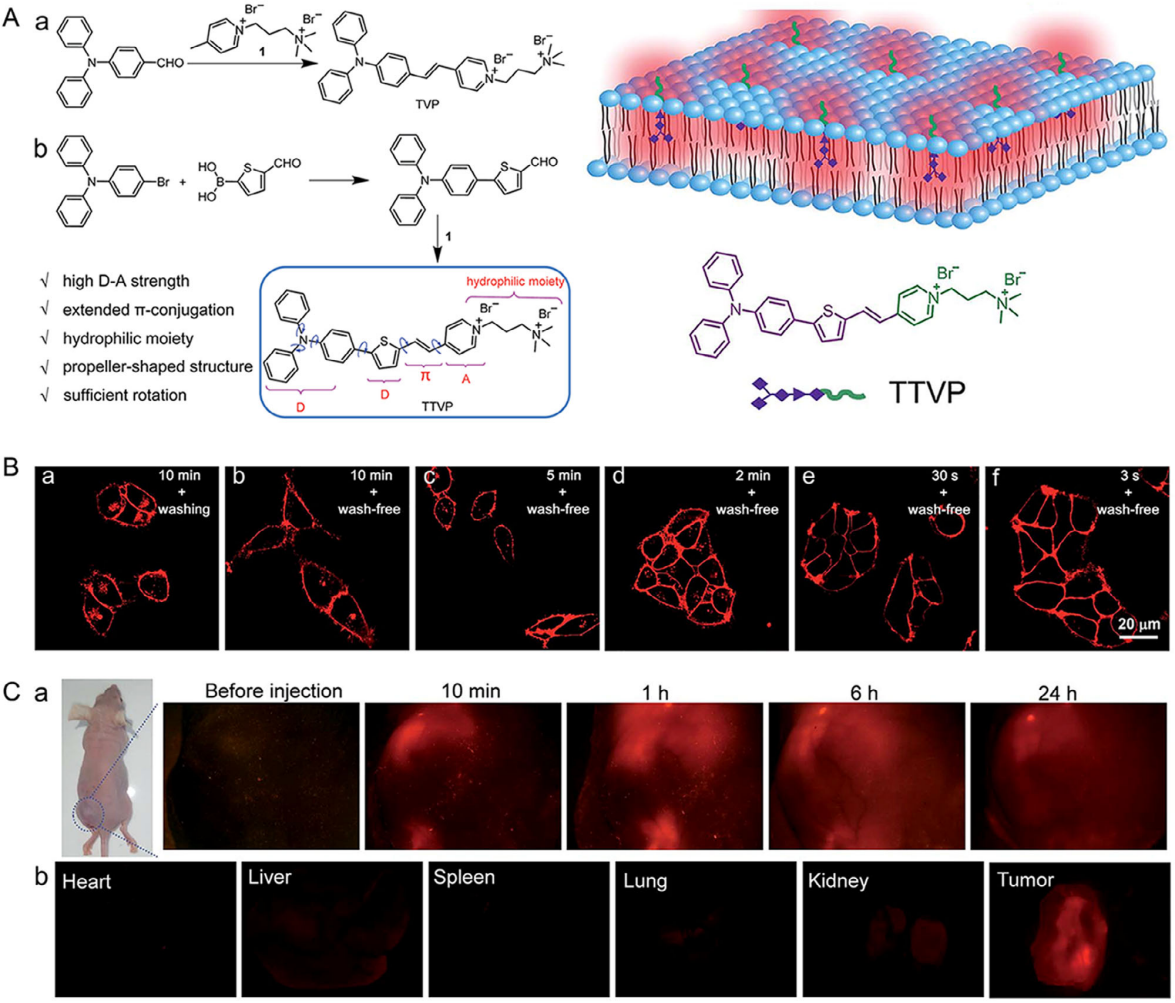
Water‐soluble and NIR‐emissive AIE molecule for wash‐free imaging of cell membrane. (A) Design rationale for water‐soluble NIR AIEgen TTVP. (B) Confocal images of living HeLa cells after incubation with TTVP (500 nM). *λ*_ex_: 488 nm (1% laser power) and scale bar = 20 µm. (C) Biodistribution of TTVP in HeLa tumor‐bearing mice after intratumoral injection of TTVP (10 mM, 20 µL) (a) in vivo and (b) ex vivo. Reproduced with permission.^[^ ^29^ ^]^ Copyright 2018, The Royal Society of Chemistry.

In view of electrostatic and hydrophobic interaction, Yu and co‐workers fabricated an AIE‐based fluorescent molecule (Pent‐TMP) for specifically targeting cell membrane,^[^ ^19c^ ^]^ which was intelligently introduced for wash‐free imaging of cell membrane in neurons, erythrocytes, and the eyeball structure of living zebrafish (Figure 2). Most significantly, Pent‐TMP was first developed to successfully visualize single erythrocytes in brain with fluorescence. This research developed an ultrasensitive wash‐free molecule Pent‐TMP for imaging plasma membranes in living biosystems, which may promote the design of fluorescent wash‐free probes and enable the extensive applications of wash‐free molecular tools in living organisms. In this study, water‐soluble TMP and AIE features of fluorescent skeleton empower the wash‐free imaging, thus providing a potential chemical structure to devise WFTs for the wash‐free fluorescent imaging in vivo. Another positive‐charged benzoperylene molecule, BP‐3, rationally designed by Yu and co‐workers, can accomplish turn‐on emission and wash‐free imaging.^[^ ^30^ ^]^ Here, positive‐charged structure can ensure BP‐3 with high affinity and long retention to cell membrane due to the stronger electrostatic and hydrophobic interaction. The photostable and amphiphilic BP‐3 functioned as a promising membrane imaging dye, which further affirmed positive‐charged probes for cell membrane imaging of wash‐free. As illustrated in Figure 3, Tang and co‐workers report a probe TTVP, which is a good example of water‐soluble and NIR‐emissive AIEgen. TTVP consists of typical hydrophilic and hydrophobic parts with an AIE moiety (Figure 3A). Indeed, TTVP can specifically and uniformly light up cell membranes with an excellent signal‐to‐noise ratio via ultrafast staining and a wash‐free procedure.^[^ ^29^ ^]^ Particularly, TTVP can generate ROS upon visible light illumination in high efficiency, which endows feasible application in photodynamic therapy (PDT) of HeLa tumor‐bearing mice. This finding promotes new tactics for the construction of efficient photosensitizers for treating cancer with PDT and accelerates the exploration of fluorescence imaging‐guided cancer therapy in further study. Cell membrane plays a vital role in maintaining cell integrity and regulating cell communication. Photosensitizers insert in cell membrane can generate ROS after light illuminating, which induces lipid peroxidation resulting in cell death. Presently, there is a rare report about wash‐free fluorescence imaging of cell membranes combined with PDT, thus this novel strategy is of the value of reference for imaging‐guided cancer therapy.

Although AIEgens mainly focus on bioimaging of cell membranes in mammalian cells and pathogens, we should not forget the relevant study on plant cell membranes. Qian's group has developed a series of wash‐free plasma membrane probes including TPD‐C12 and APMem‐1, which can be used to image multi‐plant cells with high specificity for long‐term and stanning plant cells in a wash‐free manner.^[^ ^31^ ^]^

#### 3.1.2 Organelles‐specific AIEgens

Organelles inside cells are indispensable subsystems, which conduct different functions to keep biological activities reflecting the metabolism and function of living cells. Fluctuations in cellular environmental homeostasis accompanied by disease, aging, and development, can be utilized as disease diagnosis and cell function restoration, by targeting organelles with pharmaceutics, chemical and genetic tools.^[^ ^32^ ^]^ The communication between organelles acclimatizes them to functions and changes in cellular environments,^[^ ^33^ ^]^ thus important to track the dynamic of organelles.

**FIGURE 4:**
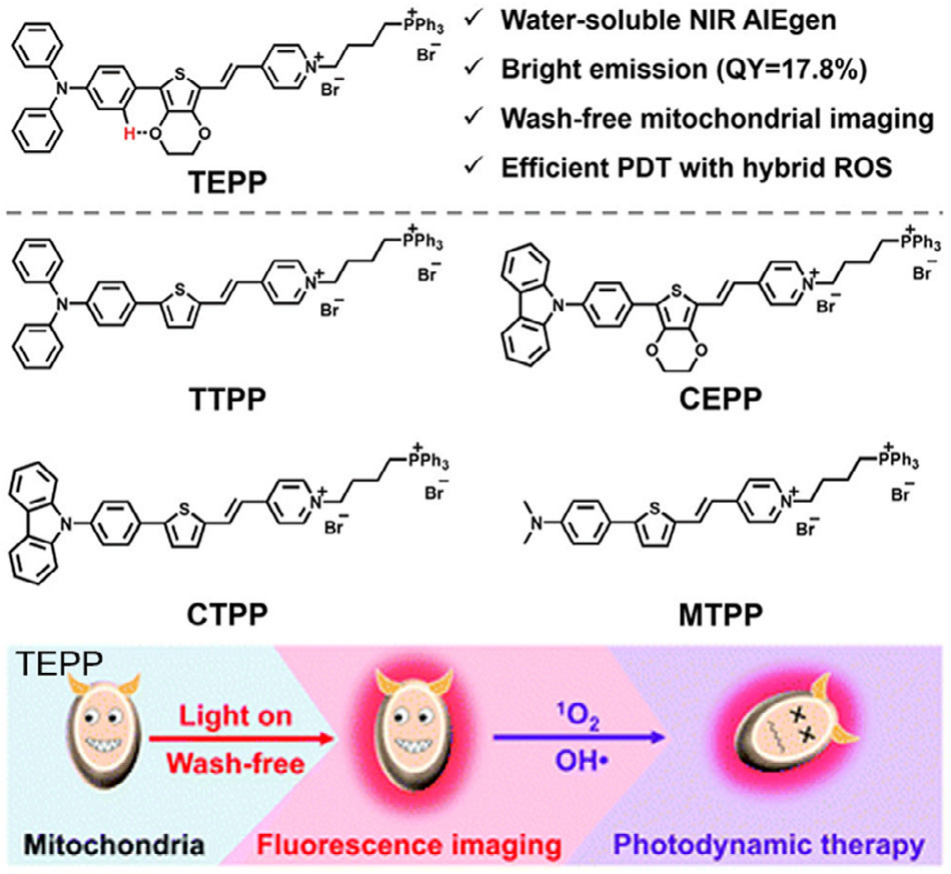
Chemical structures of different AIE‐based molecules and florescence imaging and PDT of TEPP. Reproduced with permission.^[^ ^35^ ^]^ Copyright 2022, The Royal Society of Chemistry.

*Meso*‐2‐ketopyrrolyl BODIPYs were adopted to synthesize two fluorescent molecules by Jiao and co‐workers,^[^ ^34^ ^]^ which characterized as AIE property attributed to the restriction of intramolecular rotation and J‐aggregates. These fluorescent molecules were found to specifically light up mitochondria in living cells at high biocompatibility without a washing procedure. These two fluorescent molecules also presented higher photostability than that of commercial MitoTracker Deep Red FM after being irradiated 5 min. Additionally, these two fluorescent molecules displayed deep penetration in cat skeletal muscle tissue with a satisfied signal‐to‐noise ratio. The mitochondria targeting ability, excellent photostability, wash‐free bioimaging make these probes explore mitochondria related biological process and it is valuable to study mitochondria related diseases, such as mitochondrial disorders and neurodegenerative diseases. As illustrated in Figure 4, five AIE‐based fluorescent molecules with water‐solubility and near‐infrared emission were devised and constructed by Zhu and co‐workers,^[^ ^35^ ^]^ wherein TEPP could be effectively utilized for wash‐free imaging of mitochondria with a superior signal‐to‐noise ratio. Most importantly, TEPP not only displayed bright NIR emission but also could produce ROS upon light irradiation, such as ^•^OH and ^1^O_2_, which is capable of mitochondria targeting imaging and PDT in future studies. Mitochondria, the energy factory, play a key role in regulating cell signaling pathways, respiratory circulation, and apoptosis. Mitochondria in cancer cells not only provide a large amount of ATP but also promote tumor development and metastasis through various mechanisms, such as the generation of ROS, transportation of Ca^2+^ and regulation of Bcl‐2 proteins.^[^ ^36^ ^]^ Mitochondria can be regarded as potential targets for cancer therapy. Thus, this report may provide a new insight for developing theranostic of cancers with wash‐free procedures.

#### 3.1.3 Lipid droplets‐specific AIEgens

**FIGURE 5:**
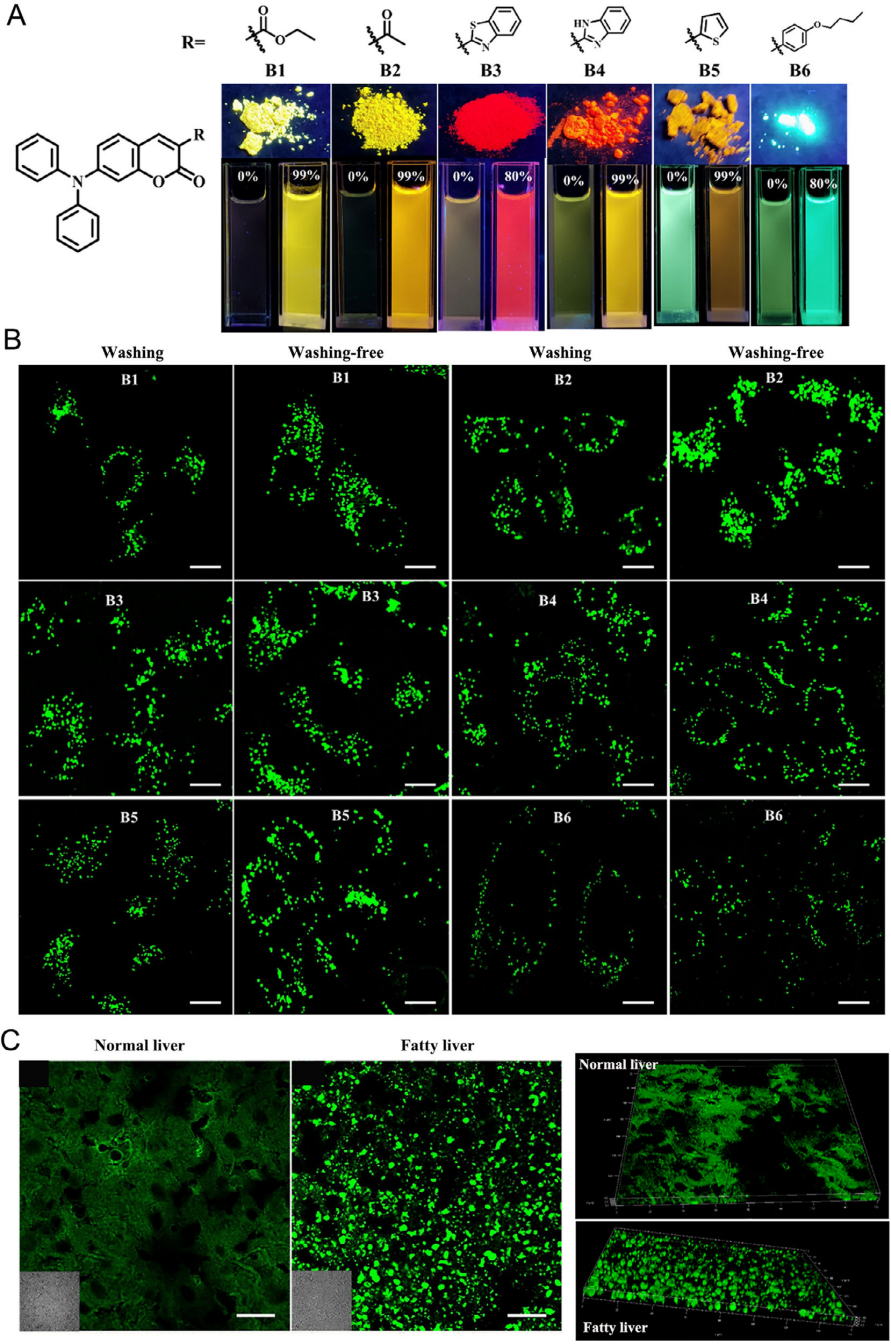
Coumarin‐based fluorescent molecules for wash‐free imaging of LDs. (A) Fluorescence images of B1–B6 in ethanol, ethanol/PBS mixtures, and in the form of powder upon excitation with a 365 nm light source, respectively. (B) Confocal images of living HepG2 cells after incubation with probes B1–B6 with or without the wash‐free procedure, respectively. For B1, *λ_ex_* = 442 nm; *λ_em_* = 520−570 nm. For B2, *λ_ex_* = 442 nm; *λ_em_* = 520−570 nm. For **B3**, *λ_ex_* = 442 nm; *λ_em_* = 530−580 nm. For B4, *λ_ex_* = 442 nm; *λ_em_* = 530−590 nm. For B5, *λ_ex_* = 442 nm; *λ_em_* = 530−600 nm. For B6, *λ_ex_* = 405 nm; *λ_em_* = 480−550 nm. Scale bar: 20 µm. (C) Fluorescence images and 3D view images of B2 in normal liver tissues and fatty liver tissues, respectively. Scale bar: 100 µm. Reproduced with permission.^[^ ^39^ ^]^ Copyright 2023, Elsevier.

Lipid droplets (LDs), composed of neutral lipids as a hydrophobic core, mainly work as a storage center of lipids, which is closely associated with many organelles.^[^ ^37^ ^]^ Meanwhile, LDs are tightly linked together with various cellular functions involving protein storage and degradation to genetic replication, which is implicated to many physiological and pathological processes, mainly including metabolic diseases.^[^ ^38^ ^]^ LDs are critical to balance the lipid species implying cellular activities, tracking LDs thus can facilitate the exploration of coordination and communication in cellular metabolism. A short series of coumarin based fluorescent molecules B1–B6 (Figure 5) were elaborately synthesized by Gu and co‐workers,^[^ ^39^ ^]^ in which B2 exhibited excellent AIE feature, ultrahigh signal‐to‐noise ratio and wash‐free property. Importantly, B2 was successfully applied to track the dynamic alterations of LDs with ultrafast staining in living cells and mouse models with fatty liver displaying excellent selectivity and resolution. Fatty liver refers to the disease characterized by mostly steatosis of large droplets, or mixed steatosis with large and small droplets.^[^ ^40^ ^]^ In addition, the enlightenment of fatty liver disease is that it commonly occurs in normal people and potential to develop as cirrhosis and even liver failure.^[^ ^41^ ^]^ As a result, real‐time monitoring LDs is of great significance for early diagnosis of fatty liver. This work has developed a promising tool for investigating the physiological changes of LDs, which is feasibility to be an effective tool for diagnosing fatty liver disease in the clinic. Additionally, a fluorescent AIE probe, TPA‐DBTD, was rationally prepared based on the dibenzothiopene skeleton,^[^ ^42^ ^]^ which can selectively visualize LDs in living cells in a wash‐free manner. As well known, lipophagy depends on the lysosomal degradation of autophagy for regulating lipid metabolism.^[^ ^43^ ^]^ Furthermore, lipophagy can regulate intracellular lipid reservoirs and the levels of free lipids in response to the extracellular nutrition supply, which plays an important role in numerous diseases, like central nervous system diseases and atherosclerosis.^[^ ^44^ ^]^ Herein, TPA‐DBTD can effectively monitor the dynamics of LDs during lipophagy in living cells and stain LDs in fungi. Thus, TPA‐DBTD is believed to be implemented to diverse living systems, especially for the diagnosis of diseases related to lipid metabolism. This kind of fluorescent tool for tracking LDs in a wash‐free manner will open the door to the exploration of lipophagy.

#### 3.1.4 Other AIEgens

Niu and co‐workers skillfully fabricated two molecules with AIE performance, DQMF‐OH, and DQMCl‐OH, functionalized with halogens (F and Cl) in the quinoline‐malononitrile chromophore.^[^ ^45^ ^]^ Surprisingly, the halogen substituted in DQMF‐OH and DQMCl‐OH, displayed absorptions and emissions with red‐shift in both solution and solid, because the strong electronegativity of F and Cl atoms can cause a narrower energy gap than that of DQM‐OH. Meanwhile, these two molecules could be employed for the fluorescence‐enhanced detection of viscosity in a wash‐free manner. This study shed new light on constructing halogen functionalized fluorescent tools due to the promoted photophysical properties by virtue of strong electronegativity. Subsequently, Chatterjee and co‐workers fabricated a water‐soluble probe with AIE features, TPE‐diBuS, which can selectively light up and realize non‐invasive wash‐free imaging of Al^3+^ in living human kidney (HK) cells.^[^ ^46^ ^]^ The reaction mechanism was proposed as ionic interactions between Al^3+^ and sulfonate groups of the tetraphenylethylene derivative in TPE‐diBuS. More attractively, the products TPE‐diBuS‐Al, reacting by TPE‐diBuS and Al^3+^, can be ensemble for tracking DNA through the interaction with calf thymus DNA (ctDNA). Consequently, TPE‐diBuS is proposed as an efficient tool for quantitatively detecting Al^3+^ with wash‐free ability and further provides tools for exploring the function of ctDNA in biological systems.

**FIGURE 6:**
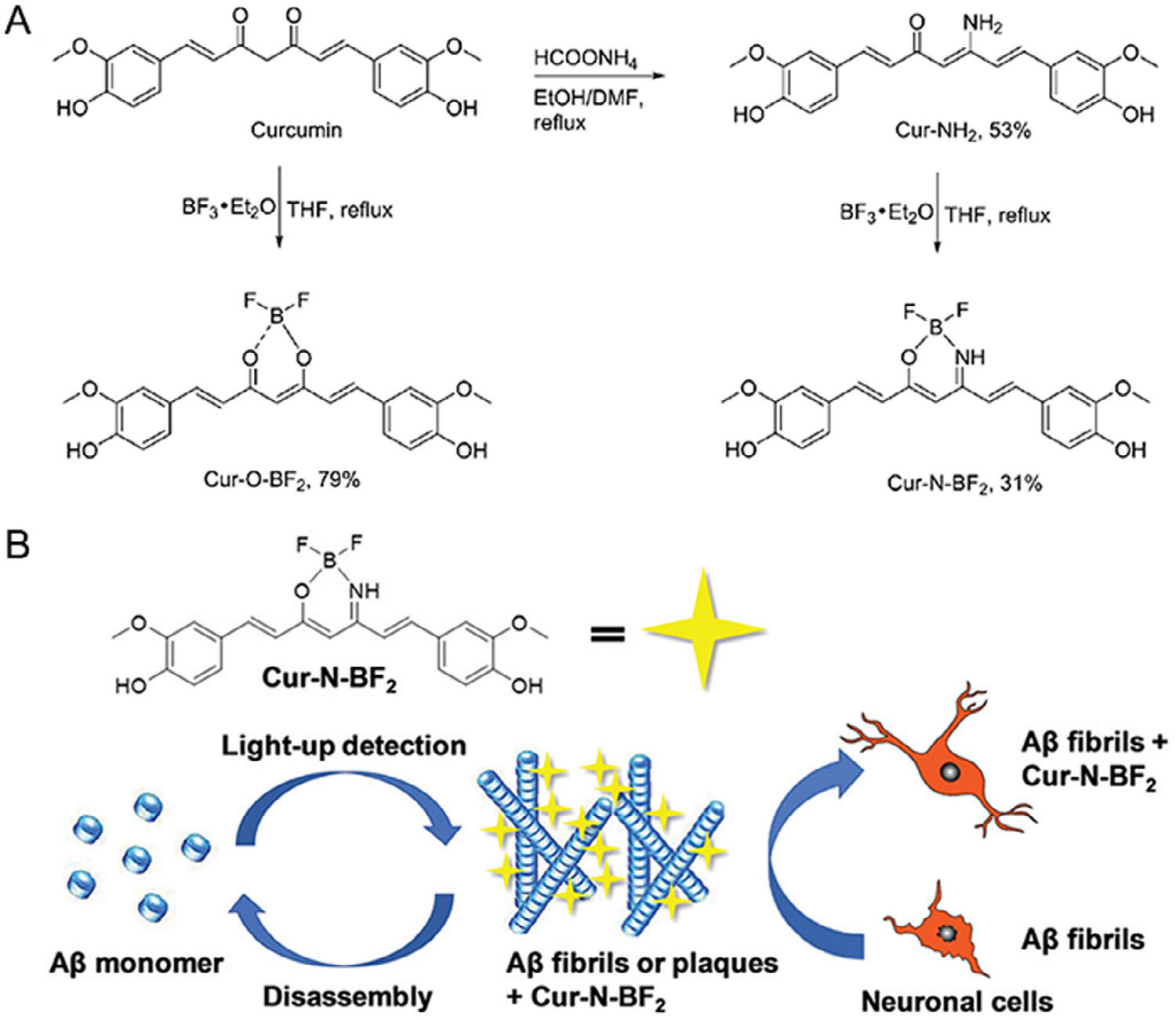
AIE based molecules for wash‐free imaging of Aβ fibrils and plaques. (A) Synthetic routes of Cur‐N‐BF_2_ and Cur‐O‐BF_2_. (B) The AIE‐active probe Cur‐N‐BF_2_ for light‐up detection of Aβ fibrils and plaques, inhibition of Aβ fibrillation, disassembly of Aβ fibrils, and protection of neuronal cells. Reproduced with permission.^[^ ^47^ ^]^ Copyright 2019, The Royal Society of Chemistry.

Epidermal growth factor receptor (EGFR) is reported to promote solid tumor growth and, thus is relevant to multiple cancers and as a crucial indicator for the diagnosis of cancers.^[^ ^48^ ^]^ Cetuximab (C225) is a human IgG1 monoclonal antibody that inhibits EGFR.^[^ ^49^ ^]^ In 2017, Tang and coworkers reported the cetuximab conjugated AIE probe mAb–CSPP for wash‐free imaging.^[^ ^50^ ^]^ mAb–CSPP showed almost no emission in normal cells but was highly emissive in specific cancer cells with EGFR overexpressed. Consequently, the current study is anticipated to encourage more exploration for screening normal and cancer cells, which is of great significance to cancer diagnosis in the clinic. In 2019, they also established a multifunctional probe, Cur‐N‐BF_2_, showing AIE performance and turn‐on fluorescence, for detecting Aβ fibrils and plaques (Figure 6).^[^ ^47^ ^]^ The turn‐on fluorescence dates back to the binding of Cur‐N‐BF2 with the hydrophobic domains of Aβ_1‐42_ fibrils to restrict intramolecular motion. Currently, neurocognitive disorders, such as Alzheimer's Disease, are a hot topic of research with the development of the aging population.^[^ ^51^ ^]^ Significantly, Cur‐N‐BF_2_ can also inhibit Aβ fibrillation, disassemble preformed Aβ fibrils and protect neuronal cells, which is thus potential to the diagnosis and treatment of Alzheimer's disease. In 2023, Sasmal and co‐workers successfully developed three cyclometalated iridium(III) polypyridyl complexes with AIE property, Ir1–Ir3, for detecting lipopolysaccharide (LPS), as well as wash‐free imaging bacteria of G^−^ *Escherichia coli* and G^+^ *Staphylococcus aureus*.^[^ ^52^ ^]^ Here, the cationic quaternary ammonium group in Ir1–Ir3, enable their aqueous solubility and their binding affinity with LPS and cell membrane. Most significantly, the detection of LPS is performed at high or lower bacterial concentrations and is also visible to the naked eye at high bacterial concentrations. On account of this, these Ir complexes are promising for the detection of bacterial contamination in aqueous samples without washing procedure, which may be of great importance to the environmental monitoring.

### 3.2 Strong donor‐acceptor system

The electron donor moieties linked with the electron acceptors by a formally π‐conjugation as the donor‐acceptor molecules, usually display the property of intramolecular electron transfer (ICT). Here, ICT is affected by the property of the substituents and the bridge, while the degree can be estimated from the electronegativities and polarizabilities of the pull and push systems.^[^ ^53^ ^]^ Generally, strong donors conjugated with strong acceptors result in strong ICT with fluorescence quenching. The molecules with ICT performance display obviously environment‐dependent optical properties, causing sensitive fluorescence changes toward the fluctuations of solvents, (micro)viscosity, and chemical species in the surrounding environment.^[^ ^54^ ^]^ Due to the special microenvironment of living systems, a strong ICT system has been usually employed to monitor polarity as a wash‐free strategy.

**FIGURE 7:**
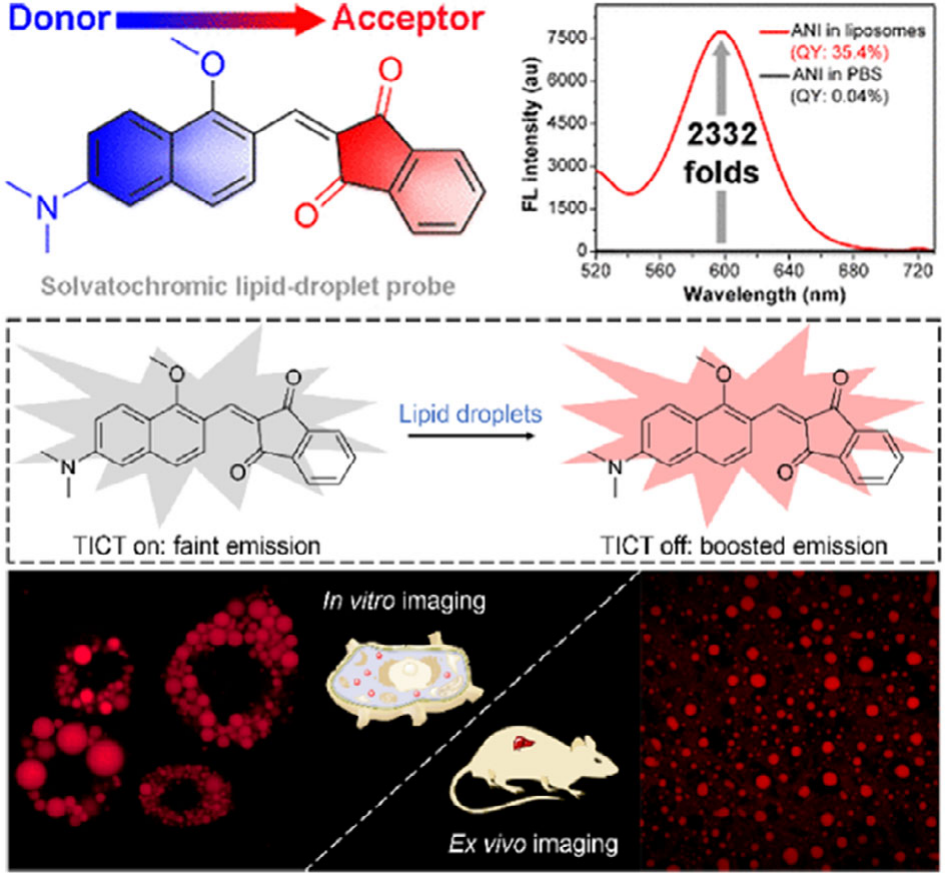
Design strategy of the solvatochromic probe ANI for in vitro LD‐specific imaging and ex vivo fatty liver disease diagnosis. Reproduced with permission.^[^ ^56^ ^]^ Copyright 2022, American Chemical Society.

Here, wang and co‐workers facilely synthesized two red‐emitting probes, L1 and L2, with donor‐π‐acceptor (D‐π‐A) structure,^[^ ^55^ ^]^ while L2 could particularly localize in mitochondria through a wash‐free procedure. Here, L2 was proved as a potential‐independent dye, it thus had stronger immobilization to mitochondria than that of commercial Mito‐tracker red. Notably, L2 was definitely identified to track the viscosity of mitochondria in living cells and also penetrate to 33.89 µm in brain tissues. This study developed a simple molecular rotor based on ICT, which is a promising tool for monitoring intracellular polarity, as well as exploring the relationship between mitochondrial viscosity and some diseases. As depicted in Figure 7, Niu and co‐workers rationally developed a lipophilic fluorescent probe ANI for the visualization of LDs without washing.^[^ ^56^ ^]^ Because of the typical twisted ICT effect, ANI exhibited almost no fluorescence in high‐polar environments, but remarkably promoted emissions in low‐polar environments (about 2332‐fold enhancement). As well known, a characteristic of hepatocytes with fatty liver is that metabolic imbalances between the synthesis and degradation of LDs lead to significant amounts of lipid deposition.^[^ ^57^ ^]^ As a result, early real‐time detection of LDs can forecast metabolic diseases, especially alcohol‐associated liver disease (ALD) and non‐alcoholic fatty liver disease (NAFLD). Here, ANI successfully revealed the excessive LDs in high‐fat‐fed guinea pig liver tissue with fatty liver. The remarkable significance of this study is that ANI is a potential tool for accurately diagnosing fatty liver.

**FIGURE 8:**
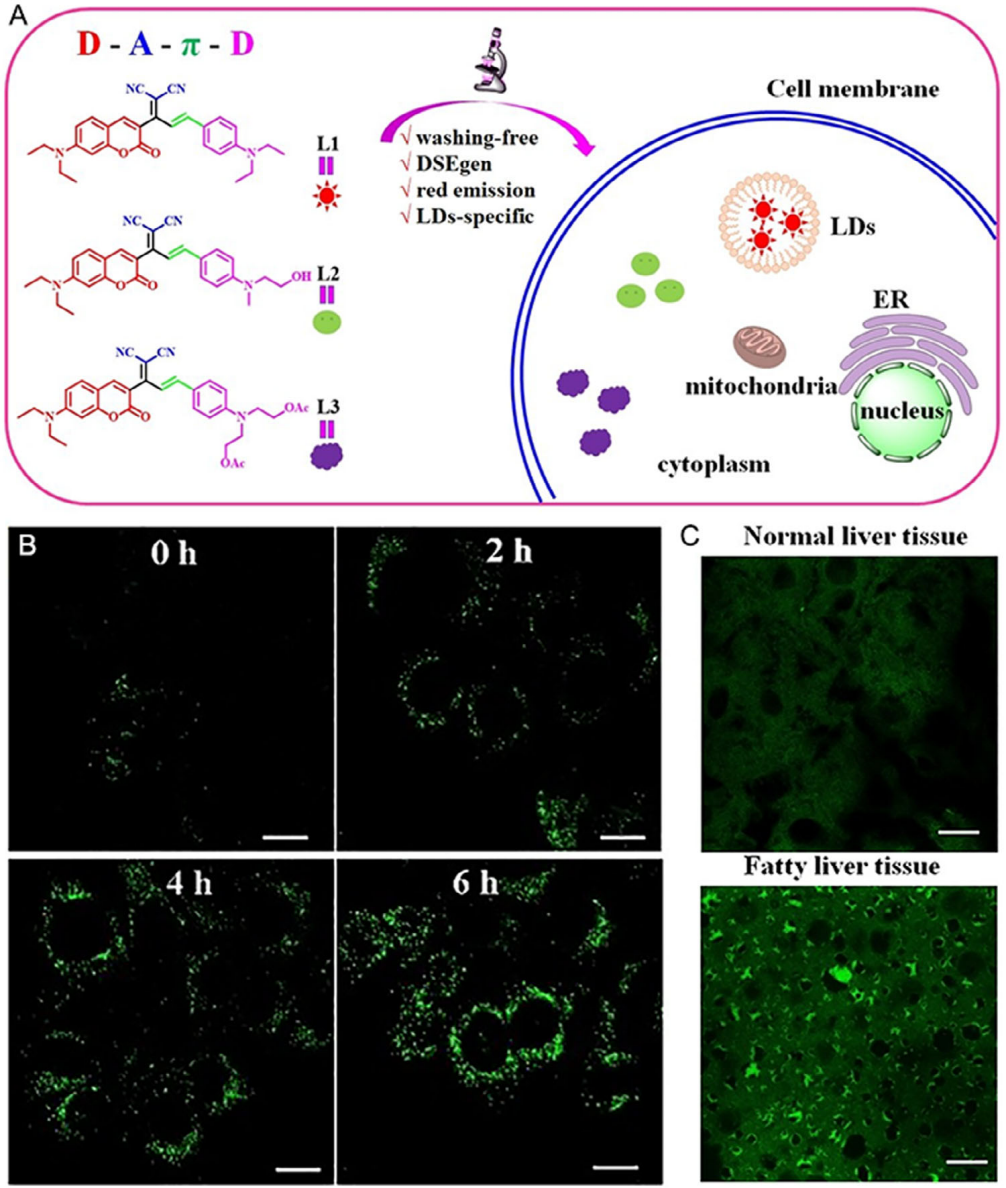
DSE molecules for wash‐free imaging of LDs. (A) Molecular structures of L1–L3 and the application for LD imaging in cells. Reproduced with permission. (B) Confocal images of HeLa cells with oleic acid treatment at different times and then incubated with L1, scale bar: 10 µm. (C) Confocal images of normal liver tissues and fatty liver tissues stained by L1, scale bar: 100 µm. Reproduced with permission.^[^ ^58^ ^]^ Copyright 2023, Wiley.

As shown in Figure 8, Ding and co‐workers synthesized three dual‐state emission (DSE) molecules (L1–L3) with D‐A‐π‐D structure for the particular detection of LDs in living cells and liver tissues.^[^ ^58^ ^]^ It should be pointed out that organic molecules that is emitted in both solution and solid state called DSE.^[^ ^59^ ^]^ This novel material has been completed in a large number of biological applications.^[^ ^60^ ^]^ The current research offers a good knowledge for the further construction of DSE active fluorescent probes. Joyfully, one of the red DSE probe L1 images LDs without a washing step, and moreover to distinguish fatty liver tissues from mouse model with NAFLD, which preserves high clarity and signal regardless of L1 concentration. This study also accelerates the development of wash‐free tools for exploring LD‐related diseases in clinics. Furthermore, Liao and co‐workers^[^ ^61^ ^]^ reasonably devised and prepared a short series of probes with D‐π‐A structure, W1‐W3, which presented a specific targeting ability to lysosomes in living cells. Herein, W1 displayed excellent stability and reversibility towards pH. Although W1 cannot accurately determine pH values, W1 was picked to qualitatively analyze lysosomal pH in living cells during apoptosis and mitophagy without washing. As a consequence, W1 is anticipated to be a potential tool for tracking the changes of pH in lysosomes under related physiological or pathological conditions. To our best knowledge, the quantitative detection of pH values in biology is more of significance, fluorescent pH quantitatively thus attracts increasing attention and attempt.

**FIGURE 9:**
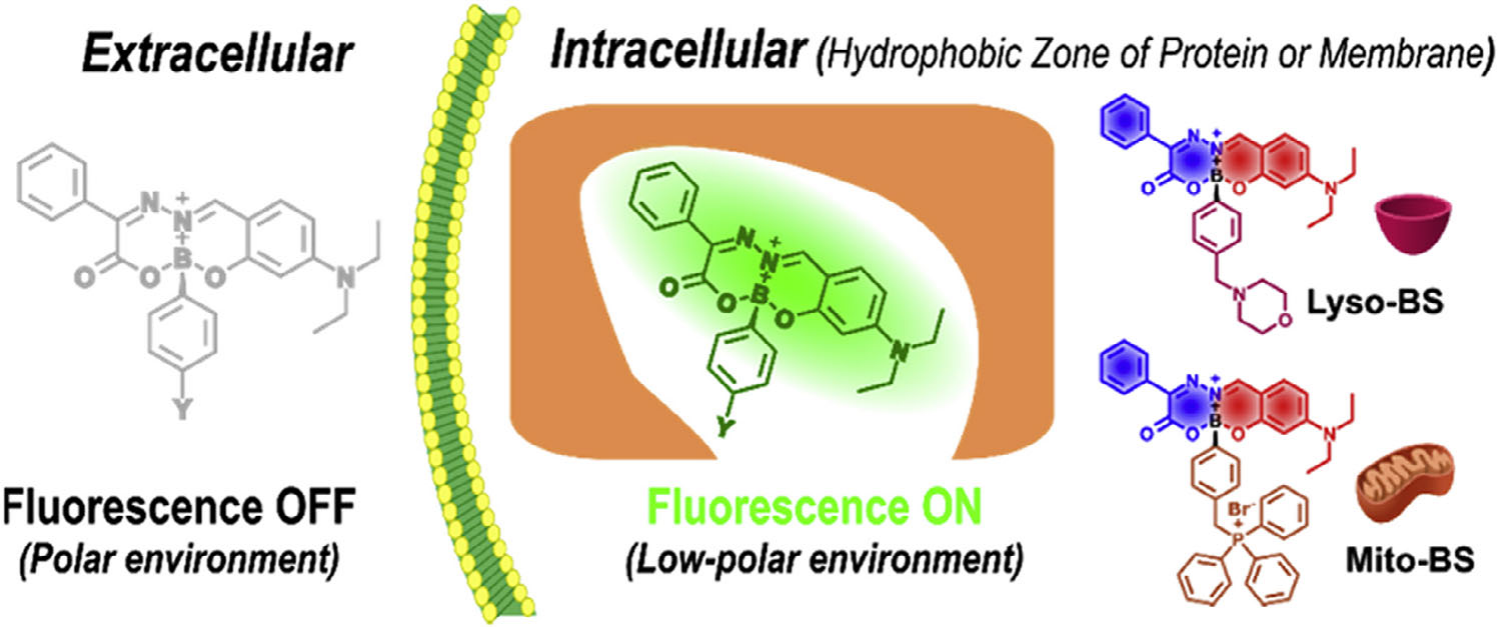
Pre‐installation of organelles targeting groups on phenylboronic acid for constructing labels of specific cellular organelles. Reproduced with permission.^[^ ^63^ ^]^ Copyright 2018, Elsevier.

Two molecules termed MOM and MOS, connected the main D‐π‐A structure with hydrophilic quaternary ammonium groups, were reasonably prepared by Feng and co‐workers,^[^ ^62^ ^]^ wherein the quaternary ammonium group can effectively increase the water solubility of MOM and MOS and also enable the cell membrane targeting property. MOM and MOS exhibited excellent cell membrane targeting ability with bright red fluorescence and without washing process. Importantly, MOM and MOS can light up cancer cell membranes with lower polarity of cell membrane than that of normal cells by comparing several cell lines, which could be employed to effectively differentiate tumors from normal tissues. Here, MOM was successfully utilized to distinguish a tumor from normal tissue in A549‐tumor‐bearing BALB/c‐nude mice, which provides new tools for distinguishing cancer cells and is potential for cancer diagnosis, due to the difference of polarity in cell membrane. As described in Figure 9, our group members rationally devised and prepared several boronic acid derived salicylidenehydrazone complexes including Lip‐BS, Lyso‐BS, and Mito‐BS.^[^ ^63^ ^]^ To obtain different organelle targeting NO_2_‐type boron complexes, targeting groups were first pre‐installed on phenylboronic acid. Among them, Lyso‐BS and Mito‐BS can rapidly target lysosomes and mitochondria as wash‐free fluorogenic dyes. The turn‐on fluorescence in lysosomes and mitochondria was triggered by the ICT effect of these complexes attributing to the polar environment. The properties of boronic acid derived salicylidenehydrazone complexes provide a potentially beneficial platform for the application of organelles colocalization.

### 3.3 Tools based on local isomerization

Local isomerization is a beneficial mechanism to achieve a turn‐on switch via tautomerization, usually breaking the conjugated system of dyes. This is particularly commonly observed in dyes of rhodamine and its analogs. For instance, the nucleophilic carboxylate group in the ortho‐position of the side phenyl, can attack the electrophilic center of the heterocycle forming spiro‐lactone.^[^ ^64^ ^]^ Additionally, the formation of spiro‐lactone rhodamines is a reversible process that is dependent on the solvent polarity, but it takes place only in a non‐polar environment.^[^ ^64^, ^65^ ^]^

**FIGURE 10:**
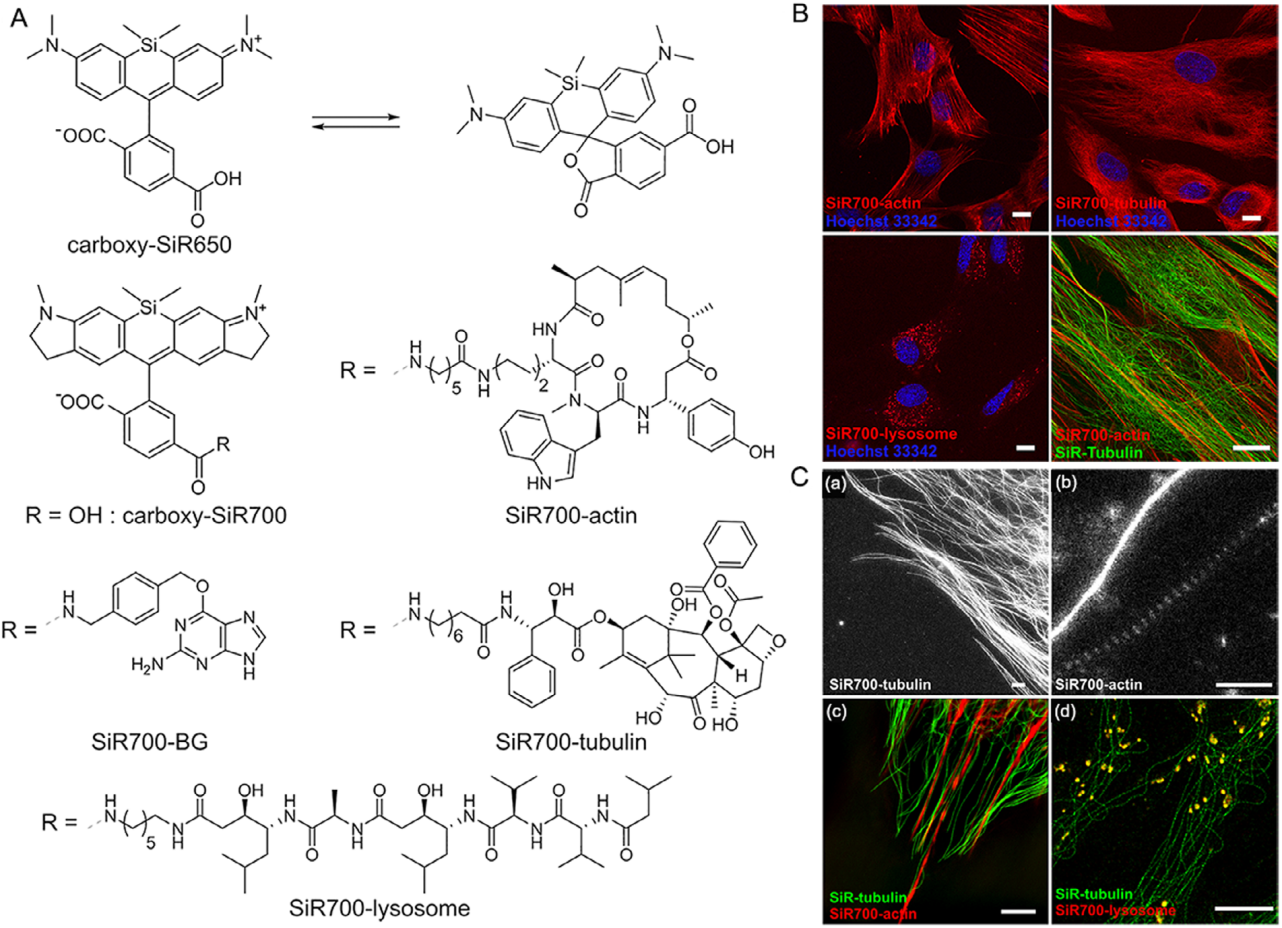
Carboxy‐SiR‐based probes for wash‐free imaging of actin and tubulin. (A) Chemical structures of carboxy‐SiR‐based probes. (B) Confocal images of human primary fibroblasts stained with the corresponding probes. The SiR700‐probe image is presented in red and overlaid with Hoechst 33342 nuclear staining in blue or SiR‐tubulin in green. Scale bars = 10 µm. (C) Super‐resolution microscopy with carboxy‐SiR probes. (a) STED nanoscopy images of human primary fibroblasts stained with SiR700‐tubulin probe. (b) STED images of primary rat hippocampal neurons stained with SiR700‐actin probes. (c) Two‐color SIM nanoscopy of human primary fibroblasts stained with SiR‐tubulin (green) and SiR700‐actin (red). (d) Two‐color STED nanoscopy of living human primary fibroblasts stained with SiR‐tubulin (green) and SiR700‐lysosome (red). Scale bars = 1 µm (a, b) and 5 µm (c, d). Reproduced with permission.^[^ ^66^ ^]^ Copyright 2021, American Chemical Society.

Johnsson and coworkers sparked this mechanism for the application of wash‐free imaging in 2016. They scheduled to adopt silica‐rhodamines (SiR), which experience ring closing at relatively higher polarity with cell‐permeable, compatible, and fluorogenic properties (Figure 10).^[^ ^66^ ^]^ However, their conjugates with biological proteins (such as tubulin, actin, SNAP tag, etc.) exhibited open form with turn‐on fluorescence only after binding to the target proteins. This strategy can effectively improve the specificity for far‐red, dual‐color super‐resolution fluorescent imaging. Notably, the developed probes based on carboxy‐SiR700 could be employed for multicolor imaging of target proteins in wash‐free procedure ascribing to the variation of the equilibrium between zwitterion and spirolactone upon targeting proteins, which can be accomplished by both conventional and super‐resolution stimulated emission depletion microscopy (STED) with the high signal‐to‐noise ratio. This study is an excellent paradigm for the transformation of fluorophores into fluorogenic probes because of the special chemical structure of SiR, which endows carboxy‐SiR many exciting applications of bioimaging.

**FIGURE 11:**
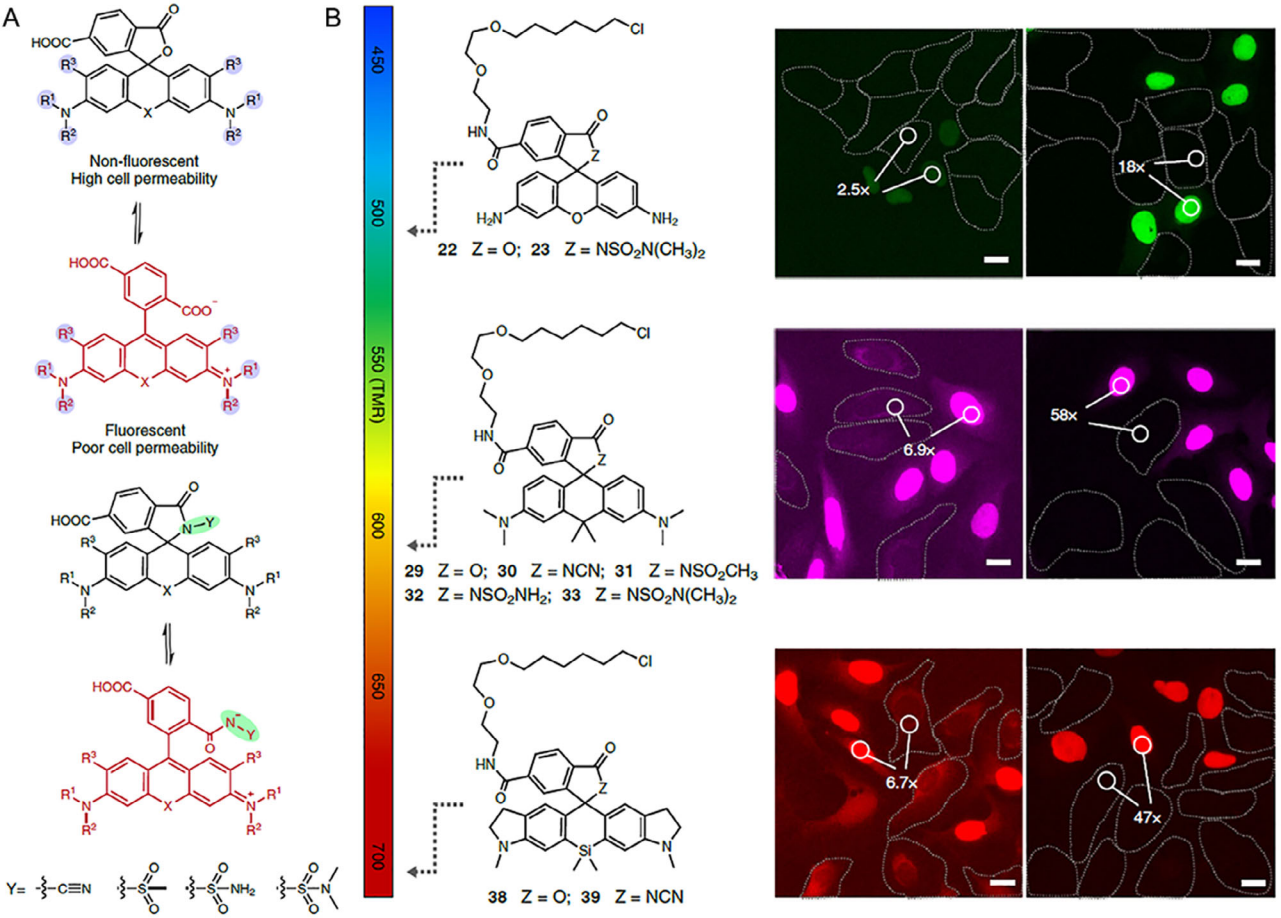
Rhodamine‐based cell‐permeable probes for wash‐free imaging of cells. (A) General structure of rhodamines and the equilibrium between the fluorescent zwitterion and the non‐fluorescent spirolactam. Y (green circle) denotes the position used to introduce EWGs to disfavor spirolactam formation. (B) Cell‐permeable probes with wavelengths that range from cyan to the NIR for no‐wash live‐cell microscopy. Reproduced with permission.^[^ ^67^ ^]^ Copyright 2020, Springer Nature.

In 2020, Johnsson et al. excitingly reported a general strategy to convert conventional fluorophores into fluorogenic probes by transforming the specific carboxyl group in rhodamines and other related fluorophores into an electron‐deficient amide (Figure 11A).^[^ ^67^ ^]^ Interestingly, the alteration did not influence the optical properties of the fluorophores but the conversion between fluorescent zwitterion and the non‐fluorescent, cell‐permeable spirolactam could be rationally tuned. They successfully synthesized a short series of fluorogenic probes in a wide range of fluorescent wavelengths and applied those fluorogenic probes for the imaging of different cellular targets without washing procedures based on this uncomplicated design principle (Figure 11B). Furthermore, probes with various fluorescent colors from cyan to near‐infrared for bioimaging SNAP‐tag, HaloTag, F‐actin, and microtubule with STED. This study makes a breakthrough in developing fluorogenic probes with great cell permeability and a low unspecific background signal.

## 4 MOLECULAR INTERACTION FOR WASH‐FREE

Molecular interaction is generally a kind of attractive or repulsive force between molecules and non‐bonded atoms. It is important in chemistry/biochemistry for protein folding, drug design, bio‐detecting, and biosensing.^[^ ^68^ ^]^ Molecular interaction has an influence on the structure/configuration of molecules resulting in the switch of fluorescence, thus playing a vital role in constructing wash‐free molecular tools. It contains four categories: (1) hydrogen bonding interaction, (2) hydrophobic interaction, (3) π–π interaction, (4) van der Waals forces interaction.

### 4.1 Tools based on hydrogen bonding interaction

**FIGURE 12:**
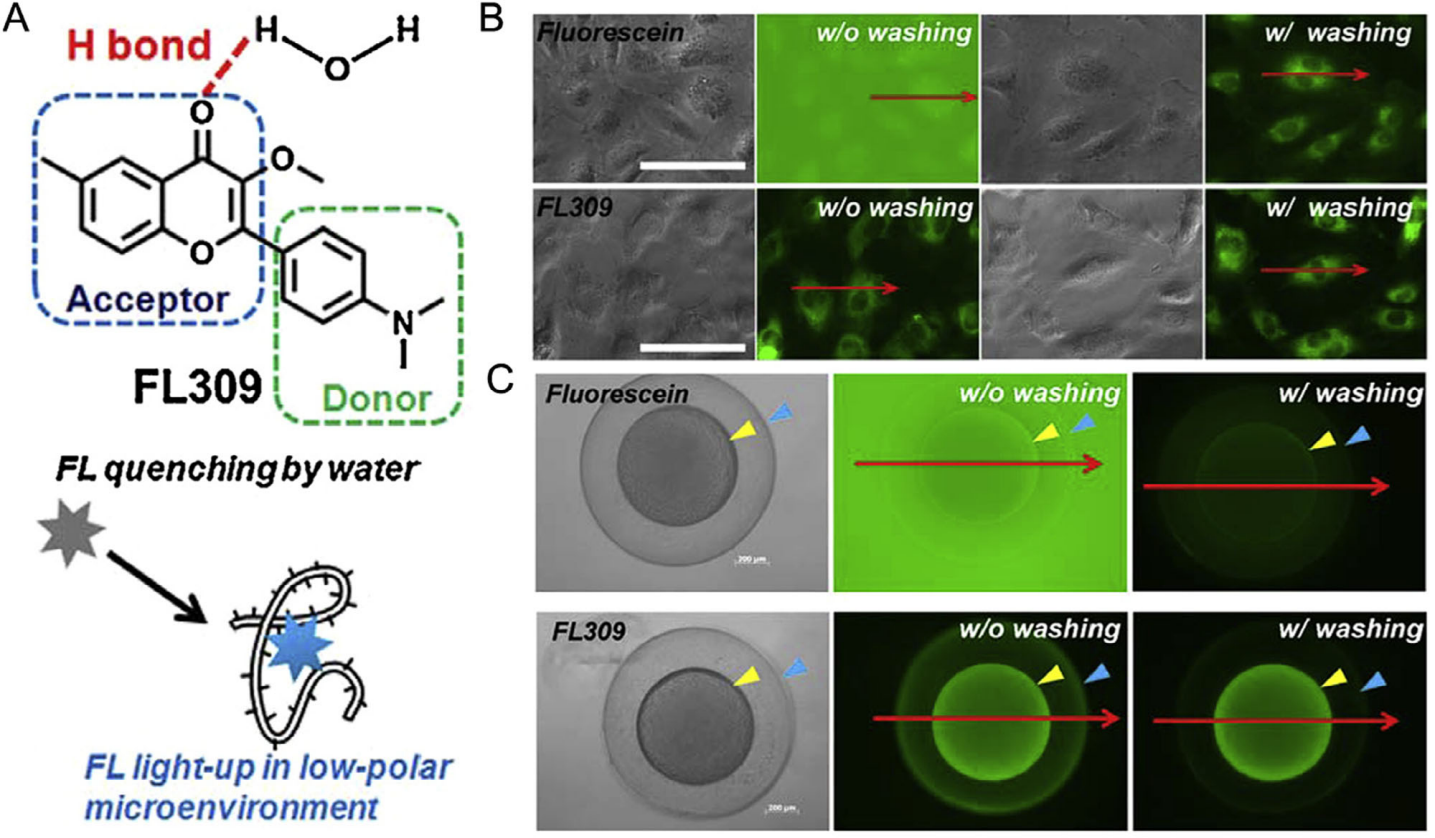
Flavonoid‐based light‐up probe for wash‐free imaging of cells and zebrafish embryos. (A) Chemical structure of FL309 and the design principle for wash‐free imaging method. (B) Fluorescent images of HUVECs stained with 5 µM of fluorescein (top) or FL309 (bottom) for 10 min before and after the washing process, respectively, scale bar: 100 µm. (C) Fluorescent images of wild‐type 4 hpf zebrafish embryos stained with 5 µM of fluorescein or FL309 for 10 min before and after the washing process, respectively, scale bar: 200 µm. Reproduced with permission.^[^ ^69^ ^]^ Copyright 2016, Elsevier.

A series of fluorescent molecules based on flavone derivatives contributed to wash‐free imaging research by using a hydrogen bond quenching mechanism. In fact, carbonyl in flavone derivatives can form hydrogen bonds with water around, thus quenching the fluorescence. In 2016, Pang and co‐workers demonstrated a flavonoid‐based light‐up probe FL309 with ICT features (Figure 12).^[^ ^69^ ^]^ FL309 was identified as non‐polar binding with Human Serum Albumin by molecular dockings. With the aid of FL309, wash‐free imaging of living cells and zebrafish embryos was successfully achieved. Subsequently, they prepared other flavone‐based probes with a lysosome‐directing morpholine unit in 2018, whose fluorescence was increased about 75‐fold in non‐polar medium than that in polar medium.^[^ ^70^ ^]^ One of these probes finally adopted to wash‐free stains of lysosomes in living MO3.13 cells for real‐time monitoring, as well as detect pH. Here, lysosomal selectivity and the increased fluorescence were associated with acid‐base interaction.

### 4.2 Tools based on hydrophobic interaction

Due to the hydrophobic interaction, a class of molecules could specifically bind to certain local regions, such as the groove in DNA, the pocket in protein, and the membrane of various organelles. The restriction of bond rotation often leads to turn‐on fluorescence as a wash‐free property, which is also a common mechanism for designing wash‐free fluorescent molecules. Seitz et al. reported a DNA‐forced intercalation (FIT)‐probe without a quencher.^[^ ^71^ ^]^ They combined the highly responsive thiazole orange (TO) nucleoside with the highly emissive oxazolopyridine analogue JO to single‐stranded TO/JO FIT probes. In general, TO/JO FIT‐probes are fluorescence quenching. In the probe‐target duplex, fluorescence is turn‐on because of torsional twisting and dye‐dye contact is prevented. This hybrid probe was utilized for the direct wash‐free imaging of oskar mRNA in *Drosophila* oocytes. Of note, TO/JO FIT‐probes can rapidly imaging RNA within a complex tissue without any intervening, which is of importance to RNA imaging avoiding cutting‐edge equipment, time‐consuming washing, and signal amplification.

**FIGURE 13:**
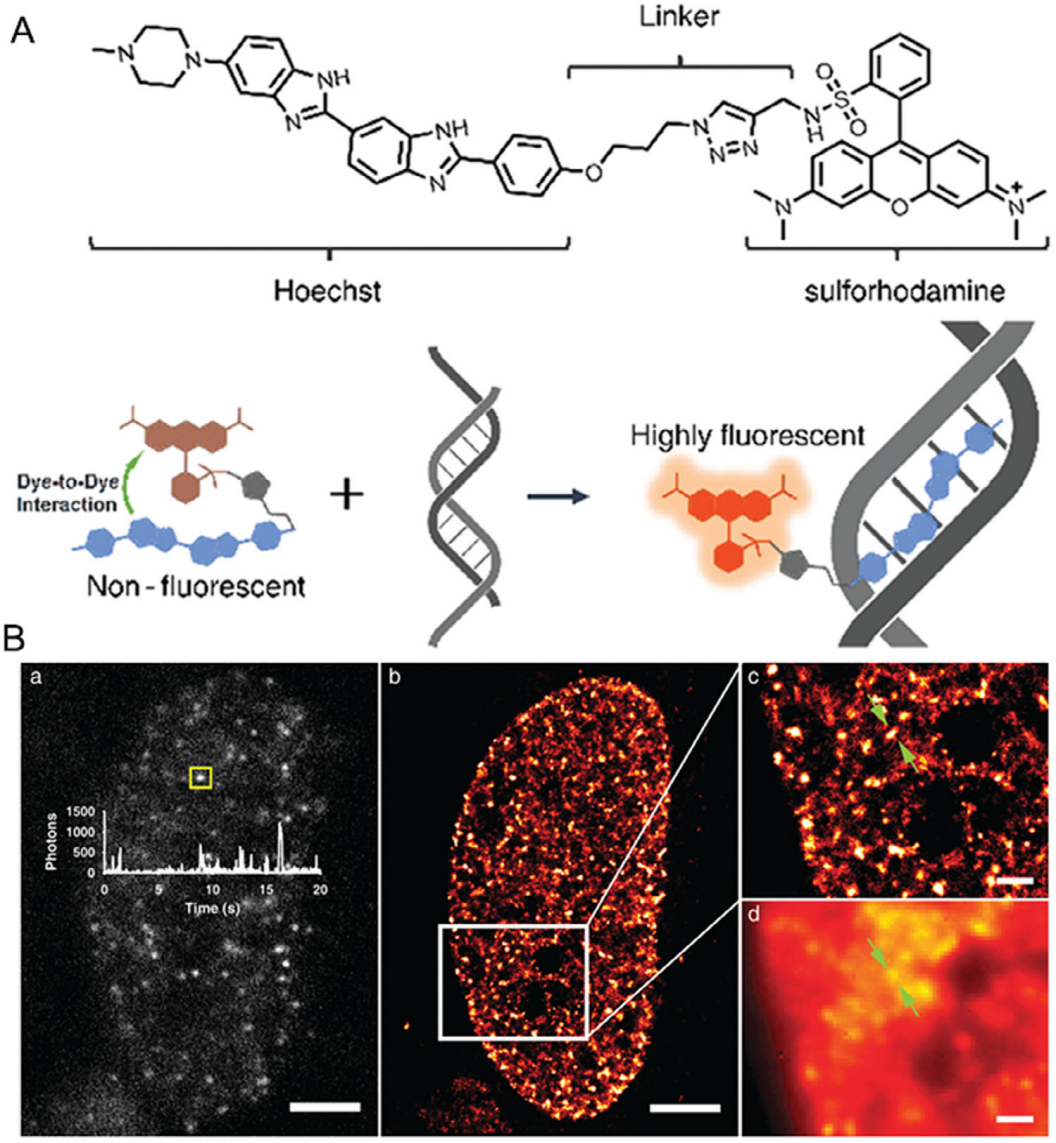
Sulforhodamine‐based probe for wash‐free imaging of nucleus DNA. (A) Molecular structure of HoeSR and Fluorescence emission mechanism of HoeSR upon binding DNA. (B) Super‐resolution imaging of nucleus DNA in live HeLa cells stained with HoeSR. Reproduced with permission.^[^ ^72^ ^]^ Copyright 2019, The Royal Society of Chemistry.

As illustrated in Figure 13, Xiao et al. presented a nucleus‐specific probe HoeSR by conjugating a sulforhodamine fluorophore with a Hoechst tag, which could label cell nucleus in HeLa cells and MCF‐7 cells displaying a wash‐free manner.^[^ ^72^ ^]^ More importantly, they successfully studied the nanostructures of the nucleus at different mitosis stages by super‐resolution imaging with direct stochastic optical reconstruction microscopy (dSTORM). HoeSR indicates exciting potential for providing structure‐related information on nucleus DNA during cell division in future studies.

**FIGURE 14:**
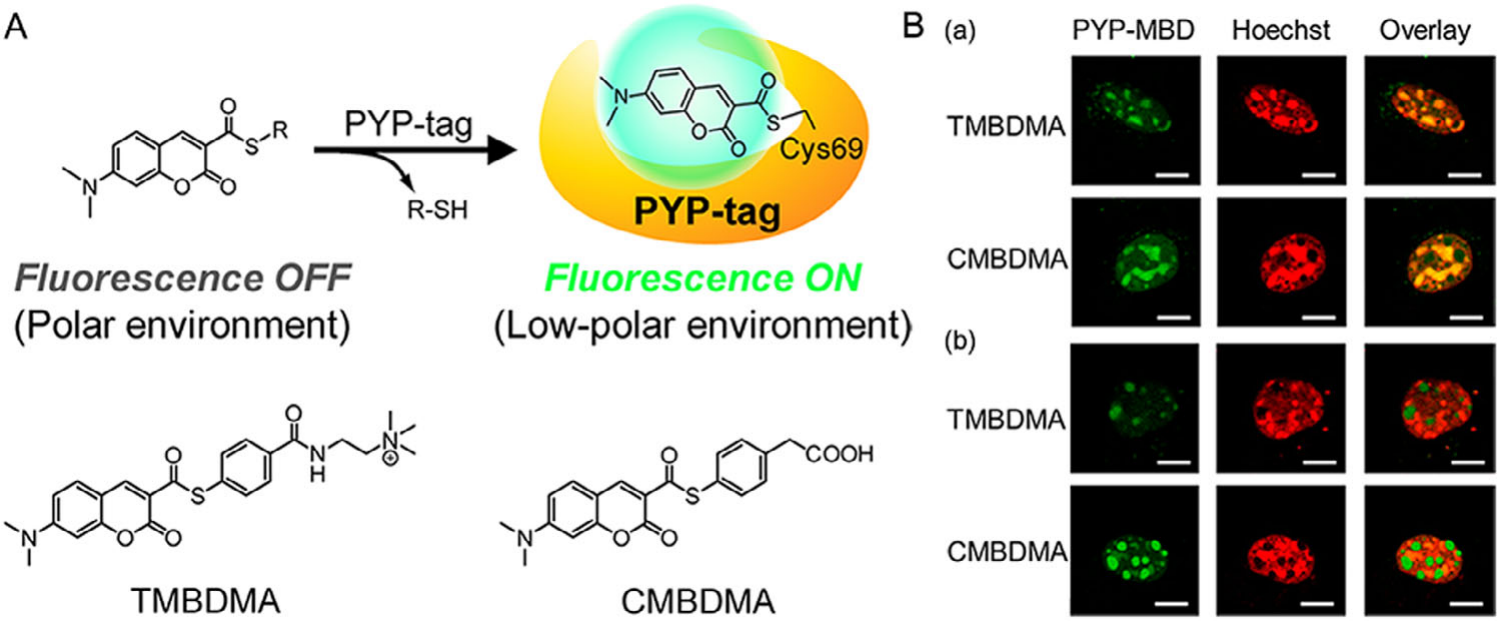
PYP‐tag molecules for wash‐free imaging of methyl‐CpG‐binding domain localization and DNA methylation. (A) Principle of labeling system based on PYP‐tag and environment‐sensitive fluorogenic probe. Structures of new probes for labeling PYP‐tag. (B) Live‐cell imaging of localization of PYP‐MBD in the (a) absence and (b) presence of 5‐azadeoxycytidine. Reproduced with permission.^[^ ^74^ ^]^ Copyright 2013, American Chemical Society.

Besides, Pang et al. reported two dyes of simple structure based on red‐emitting pyrene, wherein one of them showed fluorescence turn‐on after binding to the cell nucleus and without post‐staining washing.^[^ ^73^ ^]^ The probe displayed remarkable selectivity to stain the nucleus in both living and fixed cells on account of the pyridinium group, which has higher sensitivity than that of commercial dye (**DRAQ5**). This study is potential to update commercial dyes due to the superior sensitivity of the probe. Besides, the hydrophobic interaction mechanism is also suitable for protein‐tag technique. In 2013, Kikuchi et al. reported a hybrid probe (TMBDMA) by connecting PYP‐tag with environment‐sensitive 4‐dimethylaminocin‐namic acid thioester (Figure 14).^[^ ^74^ ^]^ TMBDMA was implemented for the wash‐free imaging of methyl‐CpG‐binding domain localization and DNA methylation, which showed the most rapid imaging feature for intracellular proteins than that of other reported fluorogenic probes (FCTP, FCANB,^[^ ^75^ ^]^ and CMBDMA). This study provides a powerful method for real‐time monitoring of the dynamic process of cellular proteins. Another hybrid probe BGSBD connecting SNAP‐tag with environment‐sensitive fluorophore 4‐sulfamonyl‐7‐aminobenzoxadiazole (SBD) was reported by Tan and co‐workers.^[^ ^76^ ^]^ BGSBD was employed for visualizing the localization of SNAP‐tagged proteins attributing to the binding of O^6^‐benzylguanine to the pocket of SNAP‐tag and adopted to the flow cytometric analysis without a wash. This fluorogenic probe is potential to developing commercial dyes for flow cytometry without washing. Furthermore, Johnsson et al. constructed a hybrid probe by connecting SNAP‐tag with the dye Nile Red.^[^ ^77^ ^]^ To our best knowledge, Nile Red is a solvatochromic molecule which highly fluorescent in a non‐polar environment while almost quenched in a polar aqueous environment.^[^ ^78^ ^]^ The hybrid probe could be utilized for wash‐free imaging of the different SNAP‐tag fusion proteins (such as the human insulin receptor) in the plasma membrane with minimal signal‐to‐noise ratio. This study provides a new ideal for establishing fluorogenic probes for wash‐free labeling SNAP‐tagged plasma membranes.

### 4.3 Tools based on π–π interaction

**FIGURE 15:**
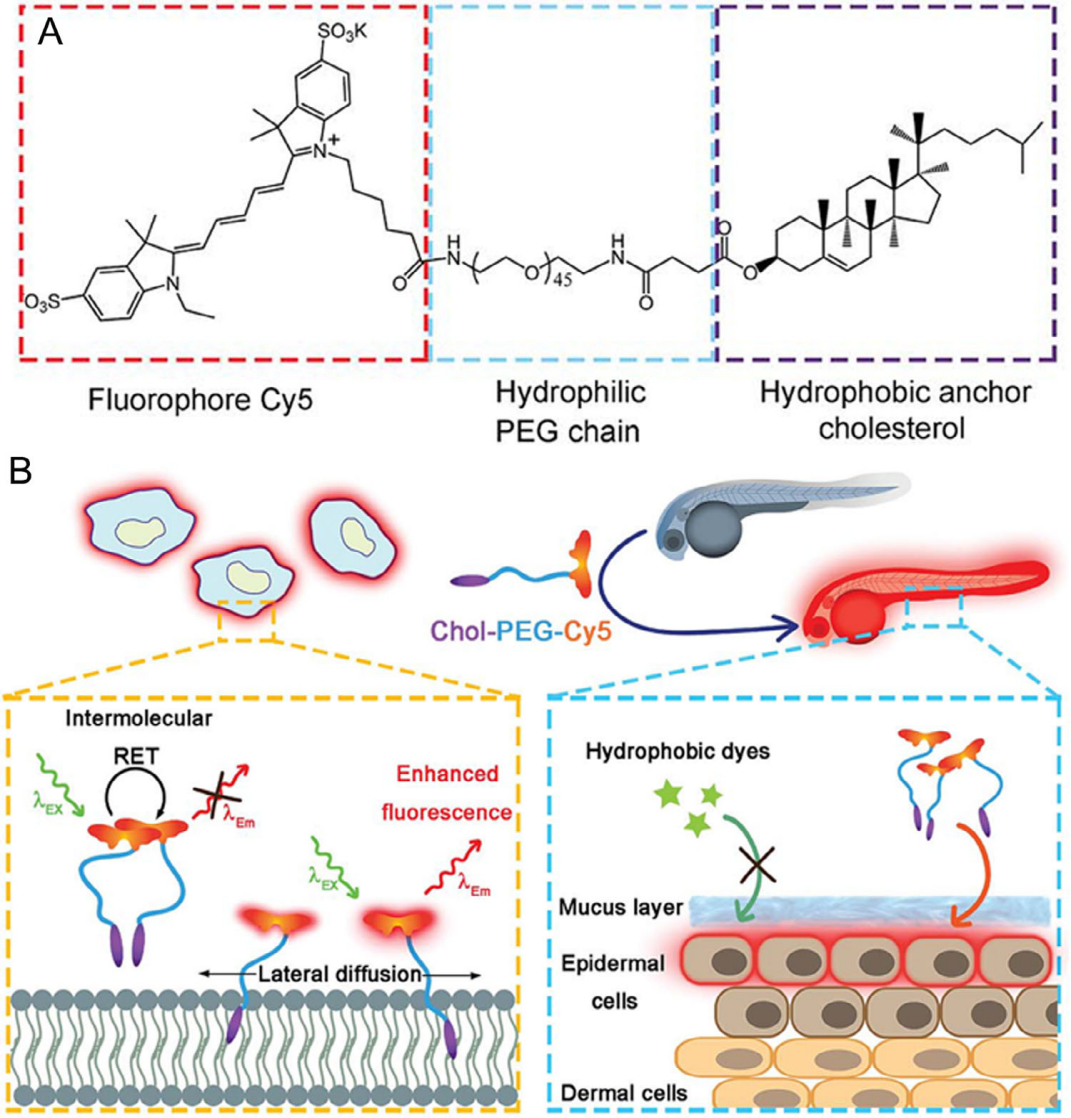
Cholesterol‐modified molecules for wash‐free imaging of plasma membrane. (A) Molecular structure of Chol–PEG–Cy5. (B) Schematic illustration of wash‐free fluorescence imaging of plasma membranes in vitro and whole‐mount fluorescence labeling of epidermal cells in zebrafish embryos using Chol–PEG–Cy5. Reproduced with permission.^[^ ^82^ ^]^ Copyright 2019, The Royal Society of Chemistry.

Generally speaking, traditional organic fluorophores are highly emissive in solutions but become weakly luminescent or even non‐emissive in aggregation, this is usually defined as aggregation‐caused quenching (ACQ).^[^ ^79^ ^]^ Most current studies are concentrated on the transformation of ACQ to aggregation‐induced emission (AIE) materials. In fact, dyes always possess flat aromatic structures thus having a strong tendency to form π‐stack mode. According to the fluorescence quenched or emitted by aggregation, aggregation‐caused quenching (ACQ) could thus be adopted to construct wash‐free molecular tools. A typical example of ACQ is the commercial probe YOYO‐1. This dimeric dye is nonfluorescent in water due to H‐aggregation, but two moieties keep a distance from each other after binding to DNA, fluorescence is thus turning on.^[^ ^80^ ^]^ YOYO‐1 has been adequately applied for sensing interactions with nucleic acids.^[^ ^81^ ^]^ As described in Figure 15, Wu et al. reported a red‐fluorescent probe Chol‐PEG‐Cy5 for wash‐free plasma membrane labeling whose fluorescence is quenched because of the intermolecular resonance energy transfer (RET) between nearby Cy5 moieties.^[^ ^82^ ^]^ The fluorescence is turned on after the dispersion in lipid bilayers. This feature renders the wash‐free in vitro cell surface imaging in a variety of mammalian cells. Although ACQ luminogens have obvious emission behavior in the free motion state such as in solution, there are still some limitations (such as strict control of concentration, fluorescence quenching results in difficult monitoring of probes, high requirement of solubility, and the like) in the development and application of organic luminescent materials, especially for biomedical applications.

### 4.4 Tools based on van der Waals forces interaction

**FIGURE 16:**
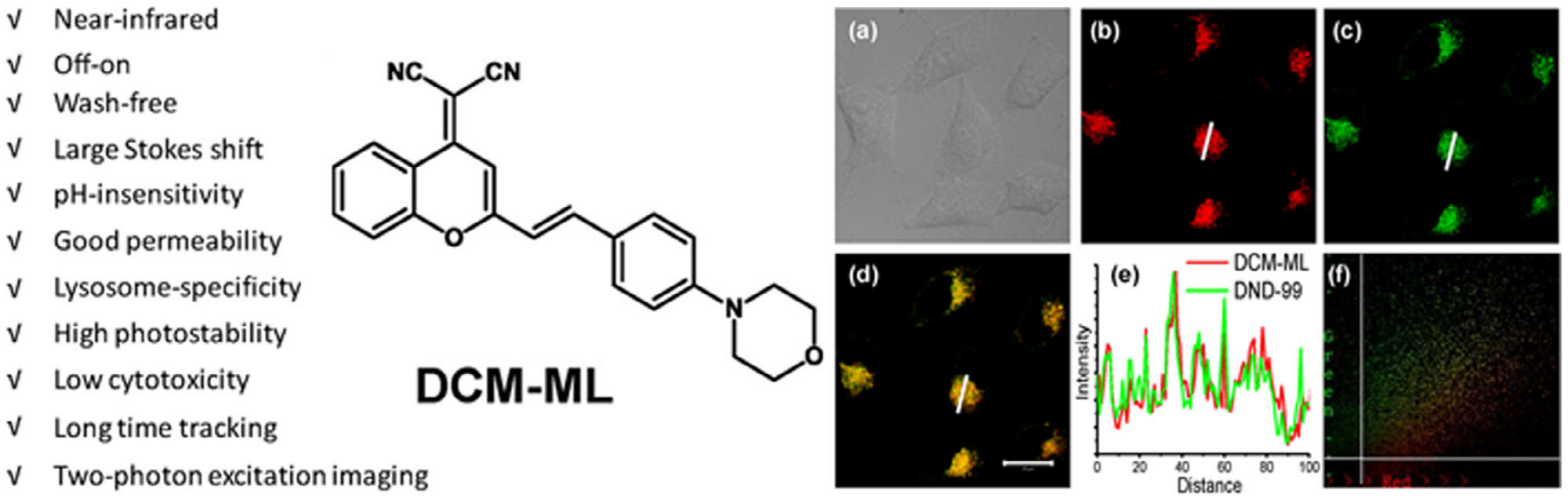
The structure of viscosity‐sensitive probe DCM‐ML and lysosome‐specific targeting of DCM‐ML in live HeLa cells. Reproduced with permission.^[^ ^83^ ^]^ Copyright 2019, Elsevier.

More specifically, van der Waals forces could be represented by fluorophore‐surrounding interactions in this review. The surrounding contains solvent, biomolecules, and other dyes; therefore, they can easily disturb the specific fluorophores to display wash‐free property. This phenomenon is often considered in viscosity‐related research. For instance, Mao et al. reported a dicyanomethylene‐4H‐pyran‐based probe, DCM‐ML (Figure 16).^[^ ^83^ ^]^ It shows very weak fluorescence in an aqueous solution (*Ф* = 0.007) but a large “off‐on” NIR emission (20‐fold) after specifically labeling lysosomes due to the high viscosity of intralysosomal milieu. More importantly, DCM‐ML can track dynamic and morphological changes of lysosome under physiological and toxicological conditions, which is potential to the study of lysosomes.

**FIGURE 17:**
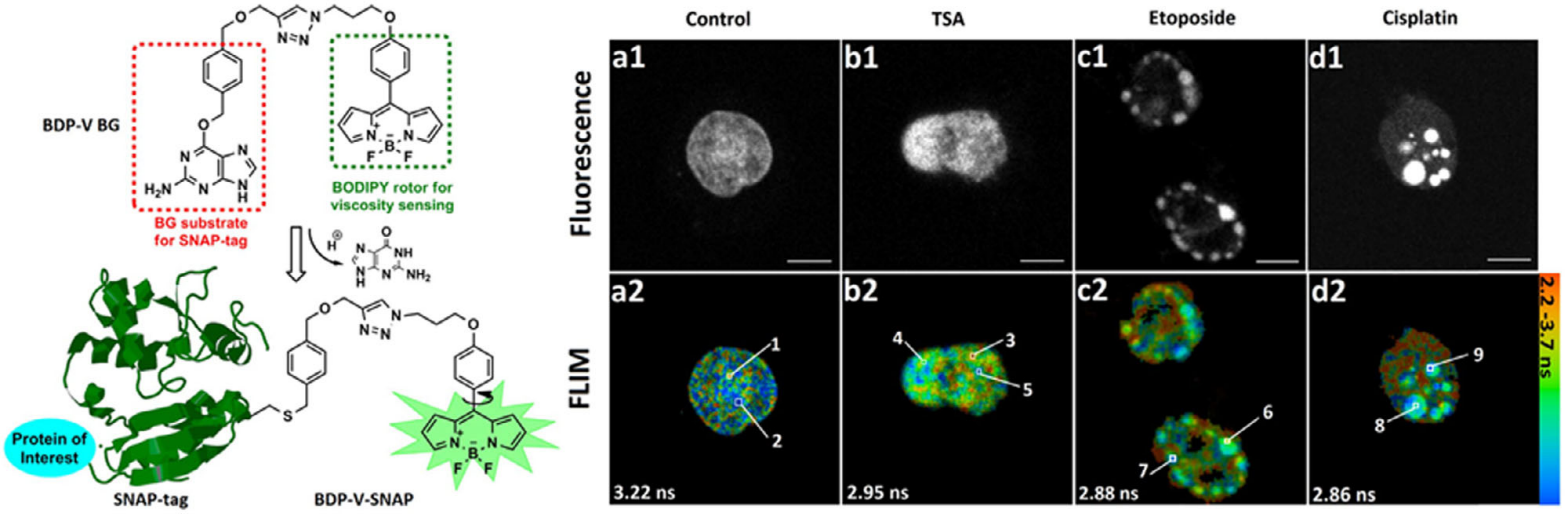
Structure of BDP‐V BG and principles of its labeling of protein fused SNAP‐tag and fluorescence and lifetime imaging of SNAP‐tag protein fused nucleus histone H2B in COS‐7 cells stained with BDP‐V BG. *λ_ex_*: 850 nm, *λ_em_*: 490−560 nm. Scale bar = 5 µm. Reproduced with permission.^[^ ^84^ ^]^ Copyright 2017, Elsevier.

Besides, Xiao et al. constructed the hybrid sensor, BDP‐V BG, by linking a BODIPY molecular rotor for sensing viscosity with *O* ^6^‐benzylguanine (BG) for specific labeling SNAP‐tag fused protein.^[^ ^84^ ^]^ As described in Figure 17, the enhancement of fluorescence induced by labeling SNAP‐tag proves BDP‐V BG as a wash‐free sensor with a high signal‐to‐noise ratio. Meanwhile, the sensor conjugated to SNAP‐tag protein getting BDP‐V‐SNAP keeps the sensitivity for the response to the changes of local viscosity with fluorescence lifetimes. Nuclear histone H2B and mitochondrial COX8A are two different SNAP‐tag fused proteins in apoptosis. Most importantly, the different fluorescence lifetime value between BDP‐V‐SNAP and BDP‐V BG, demonstrates that the micro‐viscosity near these two proteins is distinct which is proven to be relevant to the surrounding macro‐viscosity and the restriction of protein structure in apoptosis. This research offers a new perspective to develop novel wash‐free tools for exploring the viscosity of the area near the SNAP‐tag fused protein of interest.

## 5 BIO‐ORTHOGONAL REACTION‐BASED WASH‐FREE

**FIGURE 18:**
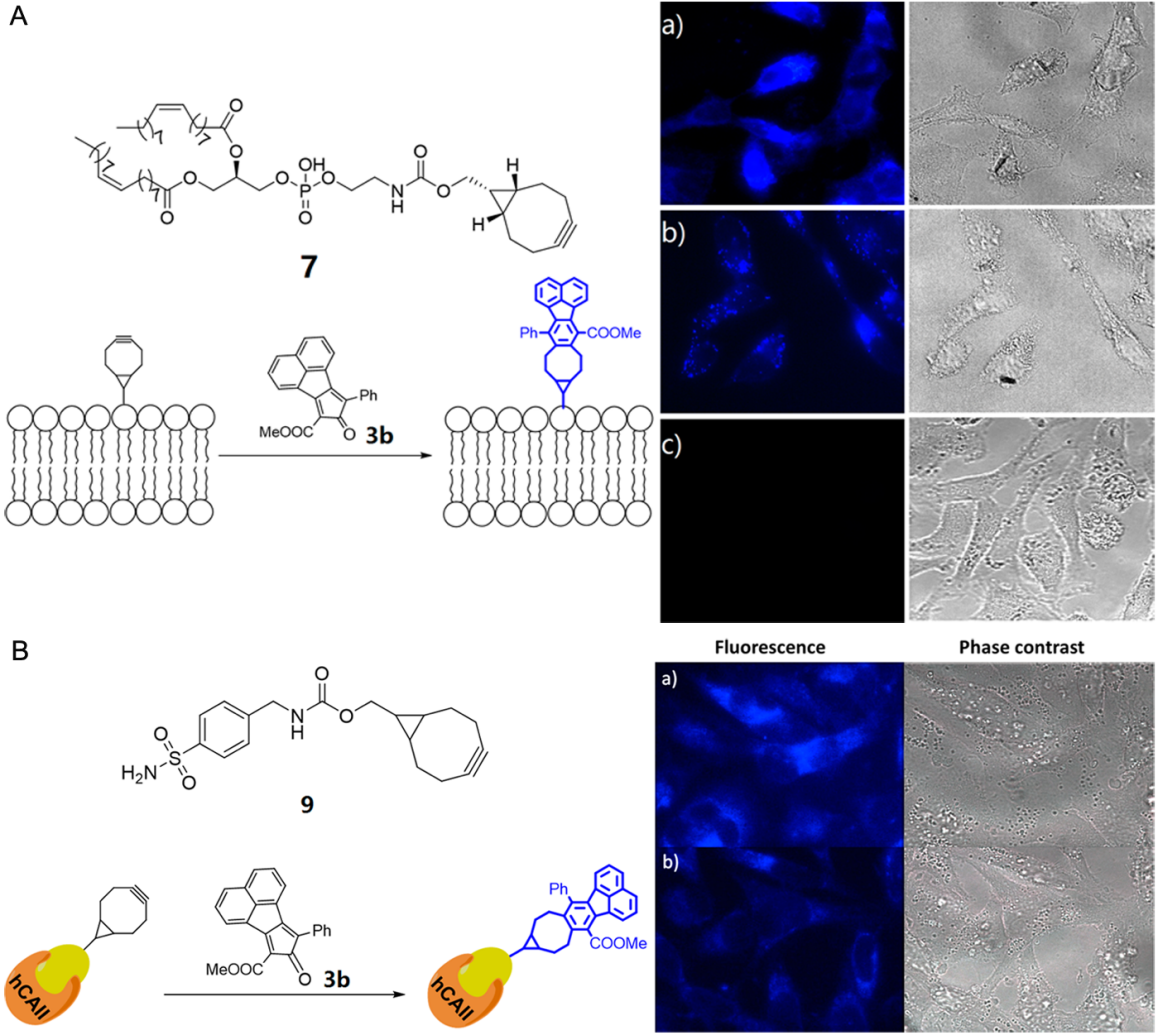
Cyclopentadienones probes for wash‐free labeling of lipids and human carbonic anhydrase II (hCAII) protein. (A‐a) Cells pretreated with compound 7 (100 µM) for 1 h and followed by treatment of **3b** (50 µM) for 4 h. (b) Cells pretreated with **4** (100 µM, without phospholipid conjugation) for 1 h and followed by treatment of **3b** (50 µM) for 4 h. (c) Cells treated with dienone compound **3b** (50 µM) only. The image was taken under DAPI channel. (B‐a) Cells pretreated with compound **9** (20 µM) for 1 h then followed by the treatment of probe **3b** (50 µM) for another 4 h. (b) Cells treated with inhibitor **8** (100 µM) for 1 h and followed the treatment of **9** (20 µM, 1 h) and **3b** (50 µM, 4 h) separately. The image was taken under DAPI channel. Reproduced with permission.^[^ ^87^ ^]^ Copyright 2017, American Chemical Society.

Bio‐orthogonal reactions that tag diverse classes of biomolecules in cells and other complex biological systems without interference from biomolecules have found widespread application in the life sciences, attributed to their high selectivity and efficiency.^[^ ^85^ ^]^ In comparison to catalyzed bio‐orthogonal click reactions, catalyst‐free click reactions are more biocompatible without the catalyst‐induced cytotoxicity.^[^ ^86^ ^]^ Catalyst‐free click reaction is a big class in the category of wash‐free imaging by transformation of the structure of the quencher group. For example, Wang et al. reported a series of non‐fluorescent cyclopentadiene probes which are highly light‐up after the labeling reaction with a strained alkyne.^[^ ^87^ ^]^ They combined compound 7 or bicyclo[6.1.0]no‐4‐yn‐9ylmethanol (BCN) modified probes with compound **3b** for the wash‐free labeling of lipids and Human carbonic anhydrase II (hCAII) protein in HeLa cells, respectively (Figure 18). Notably, wash‐free strategy based on the click reactions has the potential for high‐throughput screening of new inhibitors to the target protein in living cells.

**FIGURE 19:**
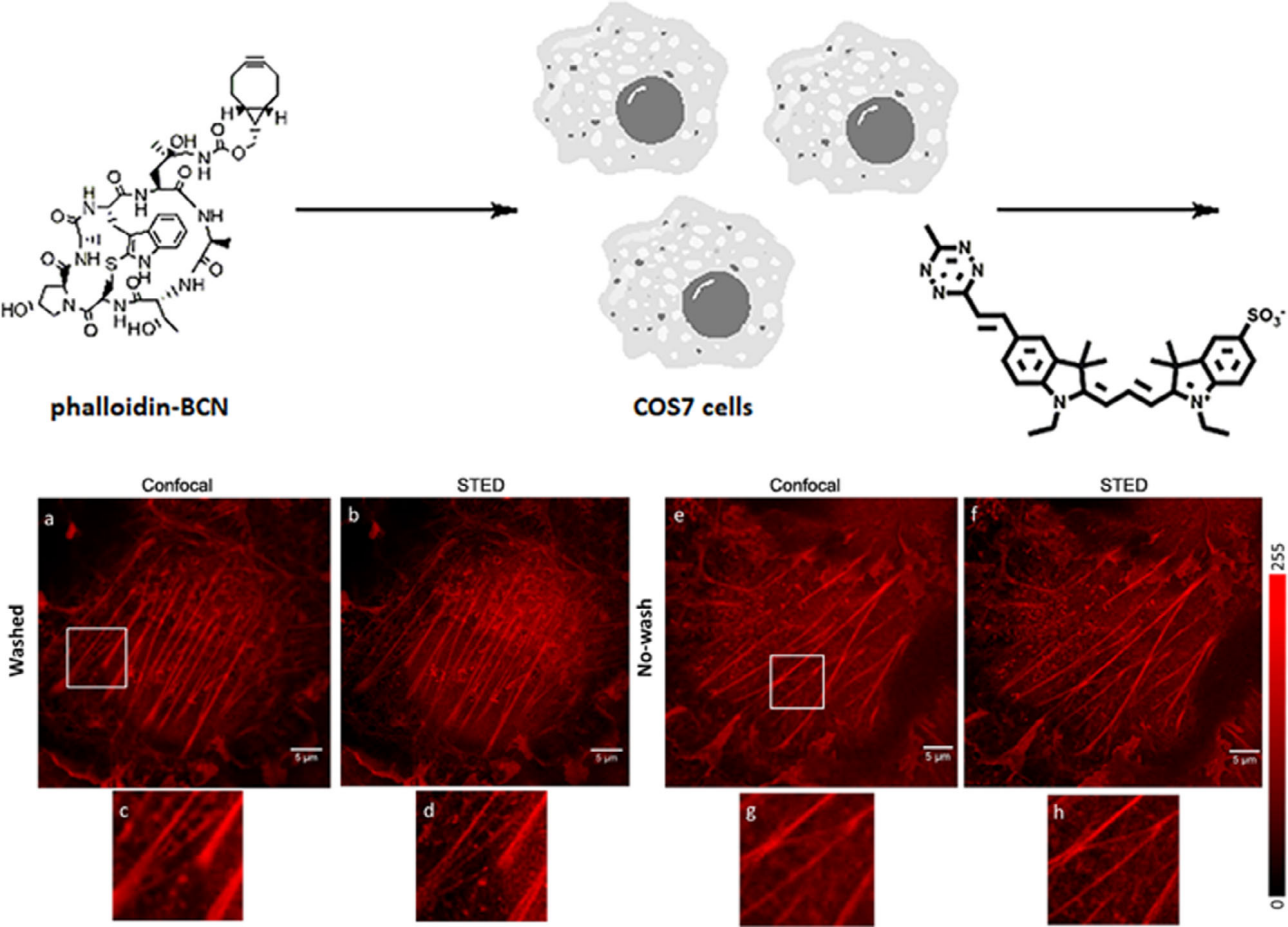
The structure of tetrazine‐quenched cyanine probes and phalloidin‐BCN, and confocal and STED images of actin filaments in mammalian COS7 cells. Reproduced with permission.^[^ ^88^ ^]^ Copyright 2018, American Chemical Society.

In addition, Kele et al. reported four tetrazine‐quenched cyanine probes and then lighted up by the click reaction with phalloidin‐BCN.^[^ ^88^ ^]^ As shown in Figure 19, this strategy is employed for confocal and STED applications under wash‐free conditions toward the imaging of labeled actin meshwork. In 2019, Taran et al. reported a series of turn‐on sydnone probes based on the efficient click reaction with cycloalkynes.^[^ ^89^ ^]^ They are suitable for wash‐free protein labeling and bioimaging inside cells with improved fluorogenic and kinetic properties. Importantly, the probe can realize super‐resolution imaging with a high signal‐to‐noise ratio.

**FIGURE 20:**
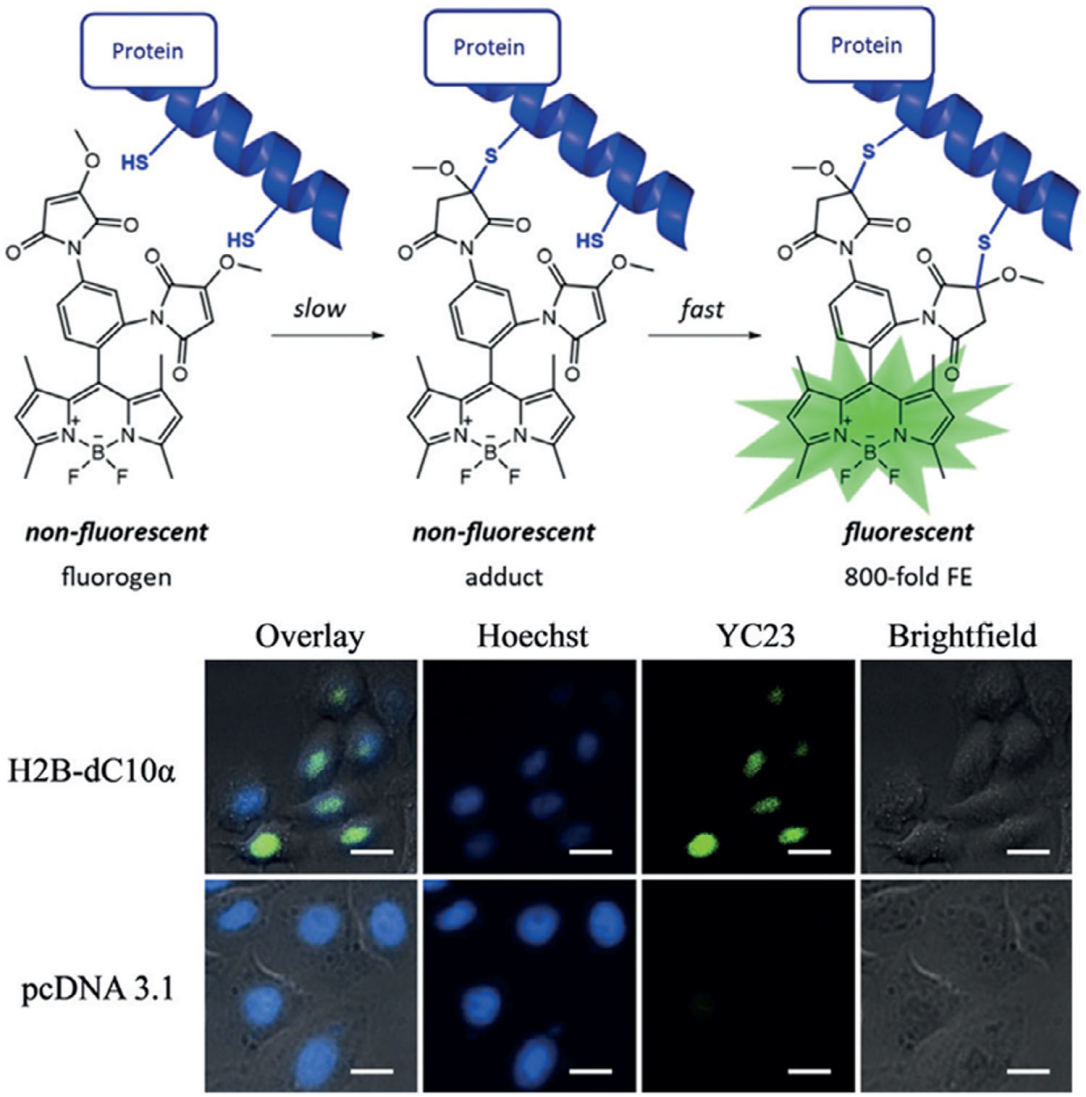
Protein labeling with BODIPY dimaleimide **YC23**, and protein labeling images of HeLa cells expressing histone H2B‐dC10α and transfected with pcDNA 3.1 (+) labeled with **YC23** (10 µm). Reproduced with permission.^[^ ^91^ ^]^ Copyright 2018, Wiley.

Meanwhile, Keillor et al. reported coumarin‐based fluorogenic probes (**YC20**) for no‐wash protein labeling.^[^ ^90^ ^]^ The turn‐on fluorescence is because of the reactivity of the maleimide groups towards the highly reactive thiols in dC10α tag. Subsequently, they reported a hybrid probe based on a green BODIPY dimaleimide derivative **YC23** and a genetically encodable peptide tag (dC10α).^[^ ^91^ ^]^ The fluorescence of BODIPY is quenched by two proximal methoxymaleimide groups, while light‐up after the addition reaction with the reactive Cys residues in dC10α (Figure 20). It was utilized to label a specific target protein in bacterial cell lysate and in live mammalian cells without washing. The significant turn‐on blue and green fluorescence in the two findings is potential for studying and labeling specific proteins of interest (POI), but the emission wavelengths of **YC20** and **YC23** are too short to image POI in deep tissues.

**FIGURE 21:**
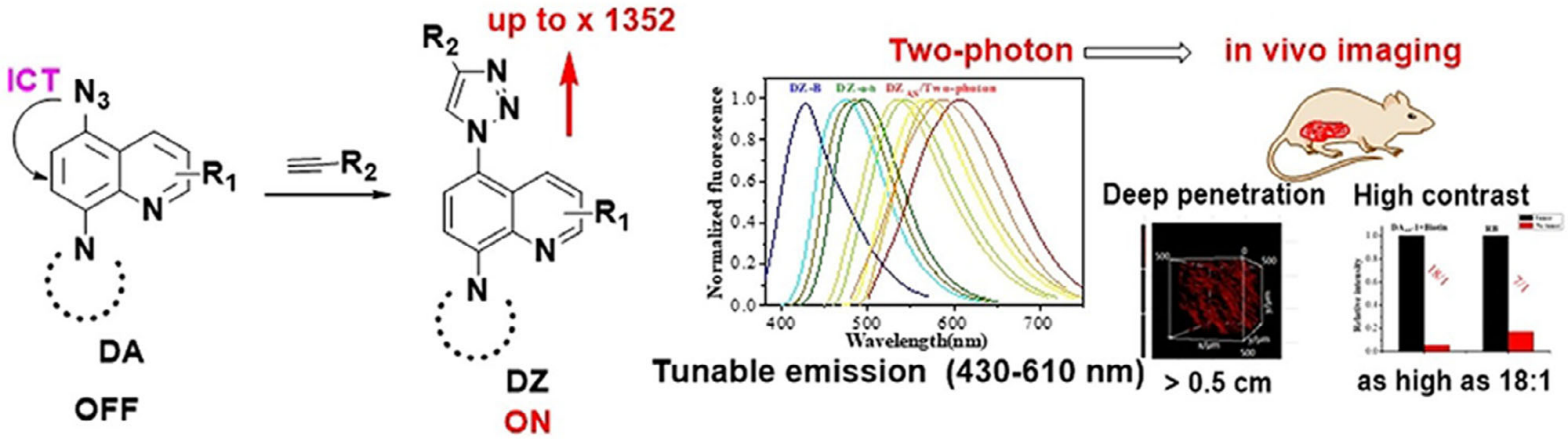
The strategy for light‐up bio‐orthogonal probes based on ICT mechanism designed by Zhu's group. Reproduced with permission.^[^ ^92^ ^]^ Copyright 2021, Wiley.

Zhu et al. reported a novel class of light‐up bio‐orthogonal probes based on azido quinoline derivatives.^[^ ^92^ ^]^ The click reaction with alkynes induces significant fluorescence enhancement up to 1352‐fold by effectively suppressing the ICT effect. These probes were successfully applied for bioimaging in live cells without washing steps, and in vivo two‐photon imaging of live zebrafish and mice (Figure 21). Importantly, they showed better resolution and higher signal‐to‐noise ratio for in vivo tumor imaging than conventional fluorophores without light‐up properties. Therefore, the bio‐orthogonal probes developed in this study will provide advantageous tools for cancer diagnosis and image‐guided therapy.

**FIGURE 22:**
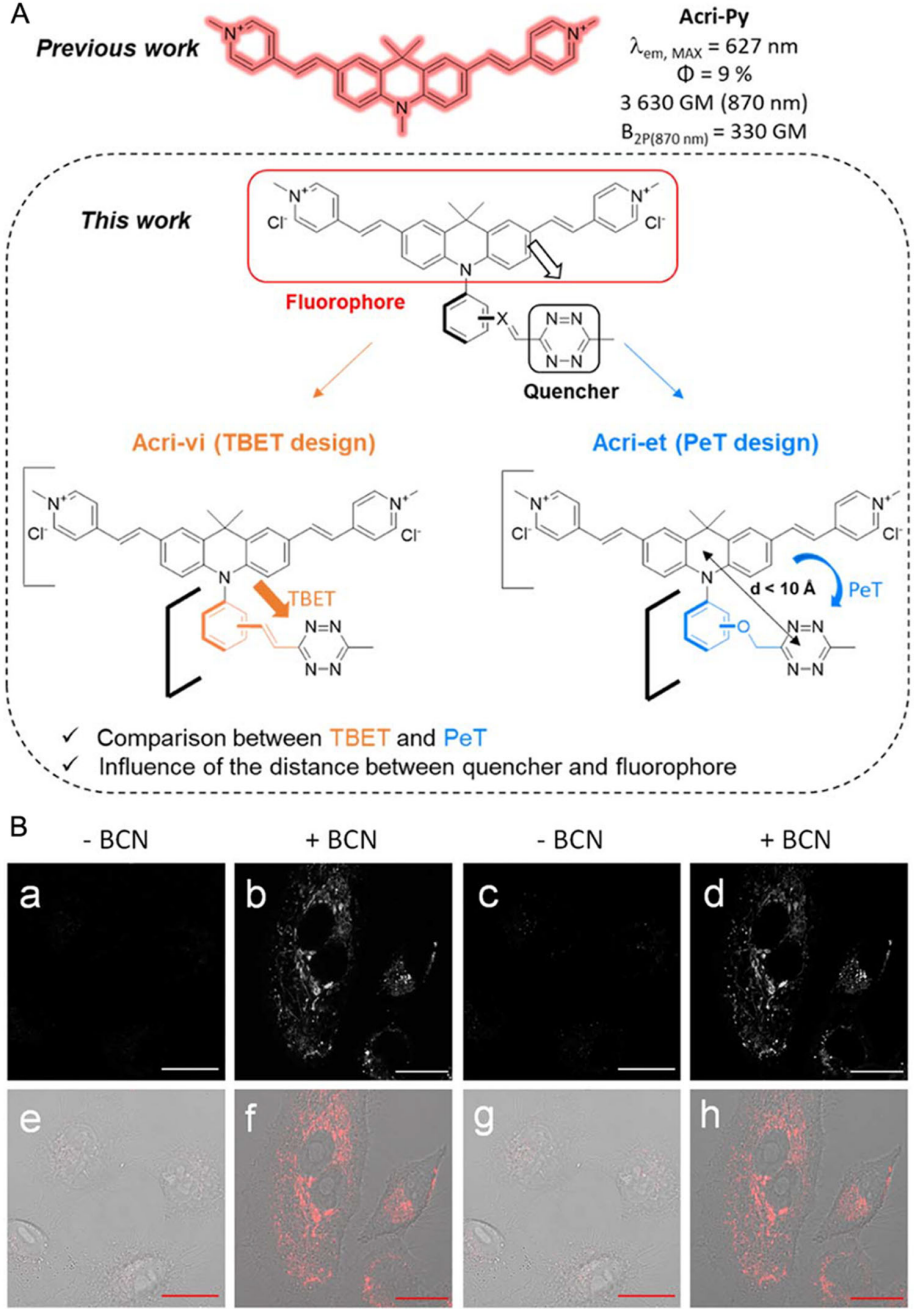
Two‐photon fluorescent probes with tetrazine quenching moiety for wash‐free imaging and protein labeling. (A) Design of the Acri‐vi and Acri‐et fluorogenic probes. (B) Confocal microscopy imaging of live A549 cells incubated with Acri‐ovi at 2 mm for 3 h and then (b,d) with or (a,b) without BCN at 50 mm for 30 min under one (496 nm, a, b) or two‐photon excitation (840 nm; c, d). (e–h) The corresponding merged images (fluorescence and brightfield). Emission slit settings: 500−700 nm. Scale bar: 20 µm. Reproduced with permission.^[^ ^93^ ^]^ Copyright 2023, The Royal Society of Chemistry.

As shown in Figure 22, Mahuteau‐Betzer and co‐workers established a short series of two‐photon excitable fluorescent probes with tetrazine quenching moiety.^[^ ^93^ ^]^ These fluorescence quenching probes displayed high turn‐on fluorescence to BCN with red emission. However, the potential Acri‐ovi exhibits a 13.8‐fold turn‐on fluorescence in living cells to monitor the reaction between BCN with no need for washing. This study developed brightness tools for compatible in vivo imaging and protein labeling.

**FIGURE 23:**
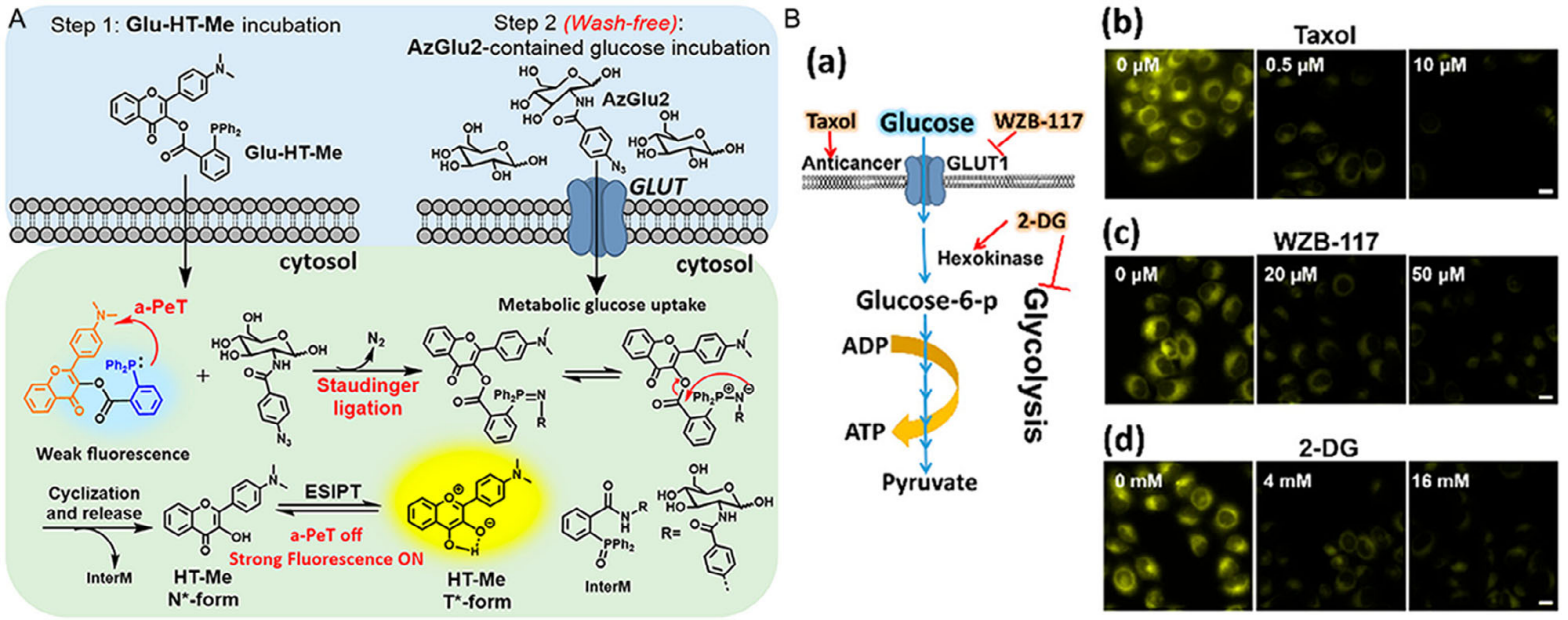
Bio‐orthogonal light‐up fluorescent probe for wash‐free imaging of glucose uptake biology and the process of cancerous aerobic glycolysis. (A) Schematic illustration of light‐up fluorescent probe presented in this work based on bio‐orthogonal Staudinger ligation for detection and imaging of cellular glucose uptake as well as chemical structures of **Glu‐HT‐Me**, **AzGlu2**, and **HTMe**. (B) Biochemical stimulation suppressing cellular glucose uptake was monitored by **Glu‐HT‐Me** using confocal fluorescence imaging. (a) Schematic illustration of anticancer agent/GLUT1 inhibitor/glycolysis inhibitor‐mediated retardation of cellular metabolism. MCF‐7 cells were incubated with various concentrations of (b) Taxol (0, 0.5, and 10 µM) for 6 h, (c) WZB‐117 (0, 20, and 50 µM) for 60 min, (d) 2‐DG (0, 4, and 16 mM) for 12 h and then incubated with **Glu‐HT‐Me** (10 µM) for 30 min, washed, and subsequently incubated with the normal culture medium containing **AzGlu2** (150 µM) for 60 min. Scale bar = 40 µm. Excitation = 437 nm, emission = 520–580 nm. Reproduced with permission.^[^ ^94^ ^]^ Copyright 2022, American Chemical Society.

A bio‐orthogonal light‐up fluorescent probe, **Glu‐HT‐Me+AzGlu2**, was successfully devised and prepared by Chen and co‐workers,^[^ ^94^ ^]^ which was effectively utilized for rapid, highly specific, and sensitive detection of glucose uptake in living cells without washing. As shown in Figure 23, the reaction mechanism was proposed as arylphosphine‐induced a‐PET effect and Staudinger ligation. In particular, **Glu‐HT‐Me+AzGlu2** was able to distinguish cancer cells from normal cells due to the high uptake of cellular glucose, and it is also capable of realizing the evaluation of glucose flux mediated by anticancer/glycolysis/transport. Glycolysis has been reported to promote tumor growth, metastasis, and chemoresistance, as well as inhibit the apoptosis of tumor cells,^[^ ^95^ ^]^ so glycolysis is of significance in tumor development and glycolysis‐targeted therapies have the potential to provide new ideas for clinical treatment of tumors. More importantly, it was adopted to monitor the fluctuations of glucose uptake in a doxycycline‐inducible K‐ras^G12V^ expression oncogenic cell system. The transformation of K‐ras^G12V^ results in mitochondrial dysfunction and a metabolic switch from oxidative phosphorylation to glycolysis, which stimulates proliferation and widespread neoplastic defects.^[^ ^96^ ^]^ The results indicated that the probe has the potential as a valuable tool to explore glucose uptake biology and the process of cancerous aerobic glycolysis.

**FIGURE 24:**
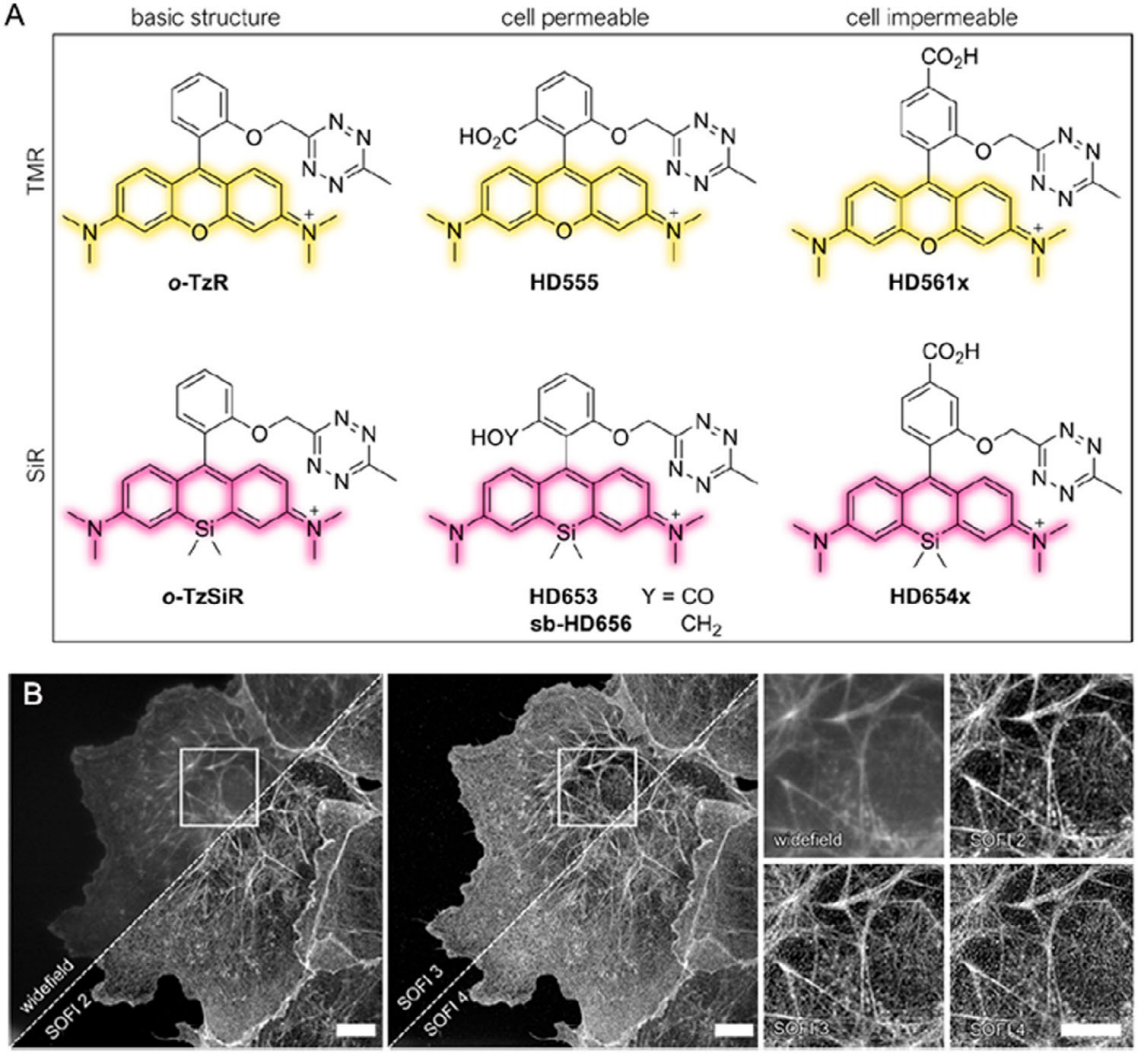
Conjugates with tetrazine and rhodamine/SiR for wash‐free imaging of cells. (A) Overview of fluorogenic TMR and SiR tetrazine probes (HDyes) with distinct cell permeability. (B) High‐order SOFI imaging of sb‐HD656 labeled f‐actin in fixed COS‐7 cells showing SOFI analysis up to fourth cumulant order. Image acquisition: 20,000 frames, 50 ms exposure time, 635 nm laser, 275 W/cm^2^. The images are representative of six cells from two independent experiments. Scale bars 10 µm. Reproduced with permission.^[^ ^97^ ^]^ Copyright 2021, American Chemical Society.

As illustrated in Figure 24, Wombacher and co‐workers reported a short series of conjugates with tetrazine and rhodamine/SiR, which exhibited cell‐permeable and ‐impermeable features.^[^ ^97^ ^]^ They all displayed highly fluorogenic far‐red emission after reacting with cyclooctene derivatives, wherein the quenching mechanism was thoroughly characterized as Dexter exchange. The applications for bioimaging in living cells were performed in combination with unnatural amino acids for multicolor labeling in a wash‐free procedure. Moreover, bio‐orthogonal labeling also achieved wash‐free labeling in living cells with super‐resolution STED and super‐resolution optical fluctuation imaging (SOFI). Here, STED and SOFI are important directions of super‐resolution technology, along with the high quality of dyes.^[^ ^98^ ^]^ These dyes provide a general motif of tetrazine‐xanthene scaffold for developing fluorogenic probes and pave the way for advanced fluorescence imaging of biomolecules with minimal label size.

## 6 ABIOTIC REACTION‐BASED WASH‐FREE

Abiotic chemical reaction represents an innovative toolkit to dissect biological processes in the context of living cells or whole organisms, such as specific fluorescent labeling, microenvironment monitoring, in situ synthesis of drugs, and removal of toxic species.^[^ ^99^ ^]^ Unlike general chemical reactions, it should proceed in complex biological systems without disturbing native physiological processes. The most prominent has been limited in practice to traditional decaging or deprotection of allyloxycarbonyl, propargyloxycarbonyl, allyl, and propargyl,^[^ ^100^ ^]^ which transformed inactivated/protected molecules to their corresponding products with activity. Although abiotic chemical reactions have attracted more attention and gradually emerged in biological applications, it is still in their infancy.

**FIGURE 25:**
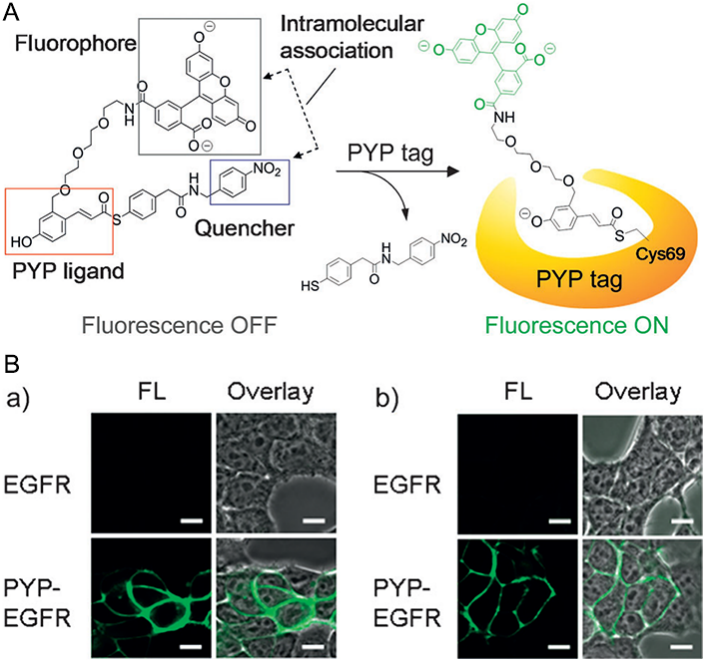
Fluorogenic probes by combination of PYP‐tag for wash‐free imaging of the PYP‐EGFR fusion protein. (A) The principle of fluorogenic labeling of PYP tag with FCANB. (B) Live‐cell imaging of PYP‐tagged EGFR on cell surfaces with the probe 5 µm FCANB (a) with or (b) without washing procedures. Scale bars: 10 µm. Reproduced with permission.^[^ ^75b^ ^]^ Copyright 2012, Wiley.

Some protein‐tag techniques belong to this category by removing the quencher with specific proteins or enzymes. In fact, the slow binding inevitably causes a decreased signal‐to‐noise ratio only improved by a complete washing procedure. Herein, the PYP‐tag, based on photoactive yellow protein (PYP), has been usually utilized for covalent protein labeling. Kikuchi and coworkers contributed a series of research works based on this mechanism. In 2012, they reported two fluorogenic probes by a combination of PYP‐tag with FCATP and FCANB (Figure 25).^[^ ^75b^ ^]^ Here, the protein‐tag‐bound strategy guarantees the ability of wash‐free for FCATP and FCANB. As depicted in Figure 25, the choice of cinnamic acid thioester in FCATP and FCANB can effectively reduce the intramolecular stacking compared with that of FCTP, thus accelerating the rapid labeling reaction. That is to say, the properties of wash‐free and fast labeling are bound to create the high signal‐to‐noise ratio. Both probes could specifically label the PYP‐EGFR fusion protein on the cell surface without the wash. Nevertheless, FCANB displayed more rapid binding capability than that of FCATP and FCTP, because of the introduction of the nitrobenzene for the preferential interaction, instead of taking precedence over the PYP ligand. This wash‐free labeling system combined with the small PYP tag offers a fluorescent tool for the imaging of rapid movement and trafficking of cell‐membrane proteins with a high signal‐to‐noise ratio.

Pan et al. reported fluorogenic probe **1** based on a fluorophore–quencher system, wherein BOBPY worked as a fluorophore and dinitrobenzene acted as a quencher.^[^ ^101^ ^]^ Bruton's tyrosine kinase (Btk) is made up of 659 amino acids.^[^ ^102^ ^]^ It has been reported as an important role in B cell malignancies,^[^ ^103^ ^]^ which is a potential target for the therapy of B cell malignancies. Here, probe **1** selectively labeled Cys 481 in the ATP binding pocket of Btk, and only be turned on by reacting with target kinase. Probe **1** was used for imaging Btk's cellular location in live cells without any washing procedure. This type of probe offers a high signal‐to‐noise ratio and is cell‐permeable and highly stable in complex biological environments, thus providing a new set of valuable tools for studying the spatiotemporal control of kinases in real‐time in living cells and even directly in real patient samples. This study has reference value for B cell malignancies in the clinic. Moreover, Xie and co‐workers reported a relebactam‐derived fluorogenic probe (RLB‐1 and RLB‐2) for covalent labeling of serine β‐lactamases (SBLs), which are the major causes of bacterial resistance to β‐lactam antibiotics.^[^ ^104^ ^]^ This highly selective imaging probe generates stronger NIR fluorescence signals upon covalently bonding to SBLs, allowing wash‐free visualization of live antimicrobial‐resistant bacteria. The exploitation of RLB‐1 and RLB‐2 further demonstrated that relebactam was a highly selective ligand to target serine β‐lactamase‐expressing bacteria. β‐lactamase is an important enzyme for hydrolyzing β‐lactam antibiotics by pathogenic bacteria inducing drug resistance,^[^ ^105^ ^]^ and the monitoring of β‐lactamases provides an opportunity for early identification of infectious diseases caused by resistant bacteria, as well as targeting drug delivery against infectious diseases caused by a resistant microbe.

## 7 SUMMARY AND OUTLOOK

Presently, the rapid development of imaging‐guided biomedical studies has improved the requirement of image quality, which results in the update of numerous tracer agents. In the matter of fluorescent imaging, the development of fluorescent dyes can be dating back to 1857, which was represented by the discovery of potassium mercury halide by James Cotton. Unfortunately, its fluorescence was not recognized at that time. Subsequently, fluorescein came into the public, which is one of the first synthetic dyes, whose derivatives are still widely applied in biology and biochemistry.^[^ ^106^ ^]^ In the early stage, the biocompatibility, chemical and photostability, specificity, along with the transportation of dyes into cells, are issues that need special attention:

(1) In general, the enhancement of lipid solubility can alter the biocompatibility of molecular fluorescent tools. Firstly, connecting with biomolecules (glucose, polypeptide, mRNA, resilin, and so on) can also obviously enhance the biocompatibility of molecular fluorescent tools, which is the most common method. Secondly, other special strategies can also improve biocompatibility. For example, Lukinavičius and co‐workers reported a neighboring group effect by putting an amide group following a carboxyl group in the benzene ring of rhodamines, to promote biocompatibility.^[^ ^107^ ^]^ Furthermore, preparation/modification with natural materials, such as collagen, pectin, chitosan, hyaluronic acid, and cell membrane, can effectively promote biocompatibility. Varlamov et al. gave a brief conclusion about pectin‐chitosan cryogels, wherein introducing chitosan can increase the degradation time and enhance adhesion to biological tissues.^[^ ^108^ ^]^
(2) Living systems are so complicated that molecular fluorescent tools will be destroyed by strong redox molecules and other bioactive molecules. In other words, these factors can be employed as special analytes for selective fluorescence imaging. Nowadays, the chemical and photostability of most molecular fluorescent tools must be evaluated before biological applications, so that they can ensure stability in living systems. Moreover, the improvement of fluorescent skeletons endows their high chemical and photostability. Some WFTs mentioned in the current review for labeling cell membranes displayed high photostability, and most are higher photostable than that of commercial dyes (DiD, Dil, and WGA‐Alexa Fluor 488). Therefore, they have the potential to replace commercial dyes.
(3) Specificity is very important for a molecular fluorescent tool, due to the non‐specific binding or reactions can yield false‐positive results thus posing limitations in biological experiments. As we know, there is usually a specific reaction site in a common fluorescent dye for reacting with analytes, thus improving the reactivity of this site can achieve high specificity. Besides, grafting molecular fluorescent tools with substrates (antibodies, DNA aptamer, inhibitors, and so forth) will also accomplish high specificity. Finally, a conditionally activated strategy can protect the dyes from damage before reacting with analysts, which can also obtain high specificity. Even so, the specificity of fluorescent tools is the direction in which researchers have been making continuous efforts.
(4) Herein, how can a dye be uptake into living cells is the first and key step to fluorescence imaging. Small organic molecules (MW < 1 kDa) usually enter cells by simple diffusion, but this is mainly decided by their lipophilicity. Thus, improving lipophilicity is an effective strategy for promoting molecular fluorescent tools in cells. Additionally, the simple diffusion is also disturbed by the anionic chemical structure of molecular fluorescent tools due to the negatively charged cell membrane. To this end, esterified precursors and cell‐penetrating peptides will be introduced to address this problem without triggering cellular toxicity or interfering with normal cellular processes.^[^ ^109^ ^]^ Furthermore, special protein/ion channels can also assist the entrance of molecular fluorescent tools. So far, the molecular fluorescent tools coming into cells is not a difficult challenge.

Subsequently, nerve‐wracking troubles, such as deep tissue imaging in vivo, weak signal‐to‐noise ratio, and emission wavelength, have gradually raised concerns. Indeed, red or NIR emission can fundamentally improve the signal‐to‐noise ratio, and it is applicable for many real‐time fluorescent monitoring. However, most molecular fluorescent tools need wash to remove the unbonded moieties in the process of using. The washing procedure can remove the uncombined fluorescent molecules to improve the quality of acquired images to some extent. However, this process may cause unnecessary interference and analyte loss, resulting in the reduction of data reliability. Therefore, there is an urgent need to develop novel molecular fluorescent tools that simplify the analytical procedure, significantly shorten the detection time, and effectively avoid unessential interference.

Herein, WFTs launch a challenge to washing‐based molecular fluorescent tools with a simple and convenient operational process, which only relies on the direct mixture of fluorescent tools and biological samples containing targets of interest. Although the wash‐free strategy derives from in vivo imaging study of protein activities, its potency quickly spread to the research of organelles, cellular microenvironments, biomacromolecules, and small species under physiological conditions in recent decades. However, many of wash‐free related reviews to date underlined more on the different applications, which to a certain extent ignored the design principle of WFTs. In this review, we have tried to discuss the design principle anticipating to providing a reference and basis for the design of WFTs. Here, we classified the design principle into four main categories:

(1) Chemical structure: AIE molecules have been extensively adopted to fabricate wash‐free dyes/probes, but they must be applied in some specific environments. Consequently, most of these wash‐free tools are utilized to monitor viscosity and distinguish biological membranes, which limits the utilization of AIE‐based wash‐free tools. Currently, organic WTFs for bioimaging are usually designed as amphiphilic structures. Whereas WTFs with poor water solubility will lead to the precipitation of probes under high‐concentration conditions. This will cause background signals caused by its aggregation, which seriously affects the accuracy of imaging. In addition, only the aggregation of molecules reaching a certain concentration can trigger the strong turn‐on fluorescence, which may result in the trouble of biosafety. In view of this, water‐soluble parts, like typical cationic substitutes, can be introduced to enable the water solubility of AIE‐based WFTs. In fact, AIE‐based WFTs mentioned in the current review, are all good at water solubility. WFTs constructed as strong D‐π‐A systems are also restricted from detecting polarity due to the π‐conjugation easily influenced by polarity. Moreover, there are fewer isomerized molecules, and they also need to satisfy the transformation of structure with special stimulates. In short, it is finite to design WFTs dependent on the chemical structures, which need further develop novel chemical skeletons.
(2) Molecular interaction: Molecular interactions play an important part in constructing WFTs. It is mainly a non‐covalent effect including hydrogen bonding interaction, hydrophobic interaction, π–π interaction, and van der Waals forces interaction. It is highly required to the chemical structures because of the formation of non‐covalent interactions. Moreover, non‐covalent interaction is prone to be easily off target and be disturbed, which thus causes troubles for bioimaging/biodetection. Further optimization should be performed to improve this design principle.
(3) Bio‐orthogonal reaction: Bio‐orthogonal chemistry is characterized as a class of chemical reactions in high yield that occur rapidly and selectively inside biological environments with minimal side reactions towards endogenous targets of interest.^[^ ^110^ ^]^ Bio‐orthogonal reactions are rationally designed selective reactions that could be employed to target biomacromolecules, small molecular metabolites, and other signaling molecules with quenching probes for visualization or identification.^[^ ^111^ ^]^ Importantly, these reactions undergo a minimum of disturbance to native biological systems.^[^ ^112^ ^]^ Bio‐orthogonal chemistry originates from a click reaction called copper‐catalyzed azide‐alkyne cycloaddition (CuAAC). At present, many other click reactions containing strain‐promoted azide‐alkyne cycloaddition, TCO (trans‐cycloctene) click reactions, have been intelligently applied in bioconjugates, biomarkers, and materials science. Consequently, designing WFTs with bio‐orthogonal reactions is an ideal strategy currently.
(4) Abiotic reaction: Moving traditional chemical reactions from flasks into living systems has attracted a keen interest from chemists. This introduces chemical conversions to living systems, which provides a universal tool for exploring and dissecting living systems. The exclusive nature of biology, metalloproteins, and enzymes can facilitate efficient multistep bioreactions. Selective transformations inside a living system can synthesize a variety of products ranging from small molecules to polymers and nanoparticles, which has been already proven in model microorganisms and human cells.^[^ ^99^, ^113^ ^]^ Although abiotic reactions have been employed to devise WFTs for imaging, therapy, and theranostics, executing non‐native chemical synthesis within the confines of living systems is still a formidable challenge due to the noninterference of the native microenvironment. Presently, the abiotic reaction is in its infancy, thus the process to be well used for constructing WFTs still has far to go.

Due to fluorescent imaging being indispensable for biological visualization, wash‐free is an increasingly popular strategy to fabricate fluorescent dyes/probes at present. It is believed to be likely to become a prerequisite feature for ingenious dyes and probes aiming at advanced in vivo imaging usages in the future. This review is expected to cause attention and thinking to establish ideal WFTs with the flexible utilization of principles now available, and on the other hand, may stimulate the emergence of novel and efficient design principles for wash‐free in large numbers.

## CONFLICT OF INTEREST STATEMENT

The authors declare no conflicts of interest.
